# Tetracenomycin Aglycones Primarily Inhibit Cell Growth and Proliferation in Mammalian Cancer Cell Lines

**DOI:** 10.3390/app152211985

**Published:** 2025-11-11

**Authors:** Kyah Birdsall, Adwowa B. S. Brako, Courtney Brown, Kendall Paige, Alexis West, Nora Schwartz, Jacob Hecht, Katelyn V. Brown, Jon S. Thorson, Khaled A. Shaaban, S. Eric Nybo

**Affiliations:** 1 Department of Pharmaceutical Sciences, College of Pharmacy, Ferris State University, Big Rapids, MI 49307, USA; 2 Center for Pharmaceutical Research and Innovation, College of Pharmacy, University of Kentucky, Lexington, KY 40536, USA; 3 Department of Pharmaceutical Sciences, College of Pharmacy, University of Kentucky, Lexington, KY 40536, USA

**Keywords:** tetracenomycins, anticancer, aglycone, cell viability, spheroid

## Abstract

Tetracenomycins are anticancer polyketides that arrest cancer cell proliferation via binding to the large mammalian ribosomal subunit near the polypeptide exit channel. The tetracenomycins are natural products that many members of the actinomycete family produce. The first goal of this study was to improve the biosynthesis of tetracenomycin analogs via metabolic engineering. The second goal was to probe more deeply into the antiproliferative activity of tetracenomycin aglycones. The tetracenomycins were assessed via several assays, including cell viability assays, clonogenic assays, and flow cytometry apoptosis assays. The data suggest that tetracenomycins C and X inhibit cell proliferation and arrest cell growth, supporting their cytostatic action mechanism. In addition, tetracenomycins C and X induced degeneration of 3D spheroid cultures and exhibited concentration-dependent inhibition of cell survival and colony formation in clonogenic assays. This work demonstrates that tetracenomycins act mainly as cytostatic rather than apoptotic agents.

## 1. Introduction

**Figure 1.**
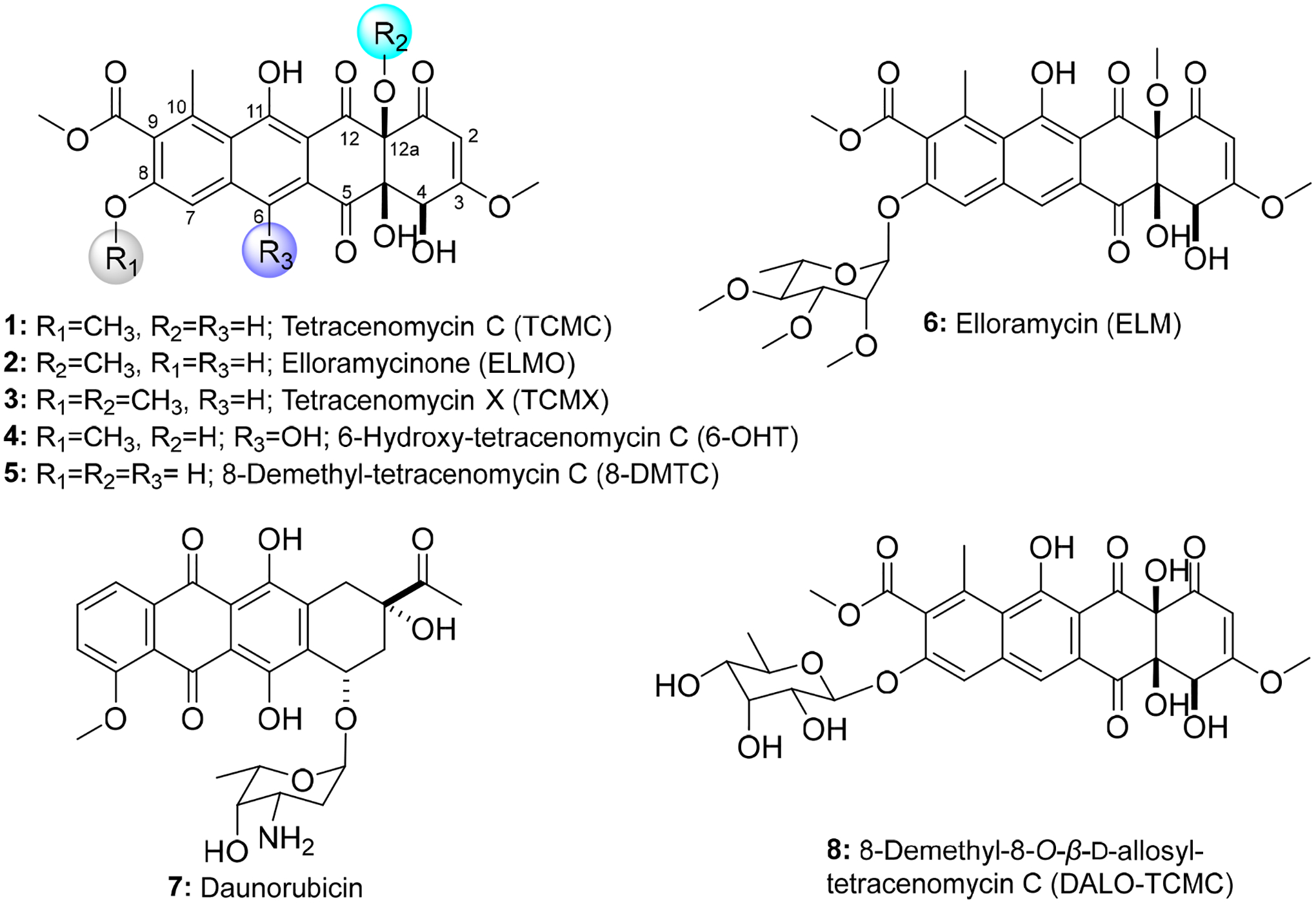
Structures of compounds investigated in this study. Tetracenomycin C (TCMC), elloramycinone (ELMO), tetracenomycin X (TCMX), 6-hydroxy-tetracenomycin C (6-OHT), and 8-demethyl tetracenomycin C (8-DMTC) represent the aglycone analogs. Elloramycin (ELM) and 8-demethyl-8-*O*-β-D-allosyl-tetracenomycin C (DALO-TCMC) represent glycosides. Daunorubicin is included as a representative anthracycline as a positive control.

The tetracenomycins are antitumoral polyketide natural products that are biosynthesized by actinomycetes [1,2]. Tetracenomycin C, elloramycinone, and tetracenomycin X are structural isomers that differ with respect to the methoxy group, which is C-8, C-12a, or both C-8 and C-12a, respectively (Figure 1) [3]. Elloramycin A is unusual because it possesses a permethylated 8-*O*-L-rhamnose sugar (Figure 1) [4,5]. 8-demethyl-tetracenomycin C is a shunt product from the tetracenomycin pathway and is the aglycone for the ElmGT-catalyzed glycosylation reaction of the elloramycin pathway (Figure 1) [6,7]. Decker and coworkers demonstrated that the UrdE oxygenase could hydroxylate tetracenomycin C at 6-position [8], producing 6-hydroxy-tetracenomycin C (Figure 1).

Tetracenomycin C and tetracenomycin X are aromatic tetracyclic natural products that exhibit the greatest potency of the tetracenomycins (Figure 1) [3]. Elloramycin A possesses significant anticancer activity despite the 8-*O*-L-rhamnose sugar appendage [3,4]. The activity of the tetracenomycins increases with the degree of *O*-methylation. Yet, some ambiguity remains regarding the structure-activity-relationships of *O*-methylation and the role of the sugar appendage in cytotoxicity. Osterman and coworkers determined that the tetracenomycins exhibit a unique mechanism of action by inhibiting peptide translation within both the 60S mammalian ribosome and the 50S prokaryotic ribosome via binding near a conserved site in the polypeptide exit channel [9]. This mechanism of action is distinct from the anthracyclines, which are topoisomerase II poisons that inhibit DNA replication, and from the tetracyclines, which bind to the 30S prokaryotic ribosome [9]. Leroy and coworkers elucidated the mechanism of action of tetracenomycin X further via inverse toeprinting and next-generation sequencing to show that tetracenomycin X inhibits peptide bond formation between glutamine-lysine motifs within the peptide exit channel [10].

Previously, we investigated the anticancer and antibacterial activity of several new glycosylated tetracenomycins with D-glucose, D-fucose, D-allose, D-quinovose, D-olivose, D-digitoxose, D-mycarose, 4′-keto-D-digitoxose sugar appendages (Figure 1) [11]. The glycosides exhibited much lower cytotoxicity than the parent natural products elloramycin, tetracenomycin C, and tetracenomycin X in the mammalian cell lines that were tested [11]. Since the parent natural products, elloramycin, tetracenomycin X, and tetracenomycin C were more active in these initial assays, we sought to conduct additional pharmacological assays to interrogate the structure-activity-relationships between the tetracenomycin *O*-methyl groups and the permethylated L-rhamnose sugar of elloramycin and in vitro cytotoxicity.

The goals of this study were two-fold: (1) to improve the biosynthesis of 8-DMTC and tetracenomycin analogs via the metabolic engineering of three novel target genes: *ssgA*, *scbr2*, and *accA2BE*; and (2) to carry out plenary pharmacological studies on the different tetracenomycin analogs to assess their anticancer potential. We successfully engineered our production host in shake flask cultures to increase 8-demethyl-tetracenomycin C titers from 275 mg/L to 854 mg/L. We next assessed the antiproliferative activity of a small library of tetracenomycins in a panel of mammalian cancer cell lines in several pharmacological assays. We performed cell viability assays, 3D spheroid culture, clonogenic assays, and apoptotic flow cytometry to assess the potency and antiproliferative activity of the analogs. We compared the activity of tetracenomycin analogs with that of daunorubicin (Figure 1), a positive control due to its potency and apoptotic mechanism of action. Tetracenomycins C and X were demonstrated to be the most potent and active in these assays, indicating that they exert their antitumoral activity via cell growth inhibition rather than apoptosis induction.

## 2. Materials and Methods

Cloning, heterologous expression, and metabolite analysis were performed as reported previously [11–17]. Mammalian cell culture and cytotoxicity assays were performed according to established protocols [18–22], with full details of media composition, transfection conditions, and analytical instrumentation provided in the Supporting Information. Plasmids are described in Table S1 in the Supporting Information. Strains are described in Table S2 in the Supporting Information.

## 3. Results

### 3.1. Metabolic Engineering of 8-Demethyl-Tetracenomycin C Production

One of the significant challenges in the combinatorial biosynthesis of polyketides is sufficient access to target scaffolds. Wong et al. identified one of the key enabling technologies of combinatorial biosynthesis of polyketides [23]. the development of chassis capable of producing high titers of polyketides via the expression of cognate polyketide synthases (PKS). With this goal in mind, 8-demethyl-tetracenomycin C could be a suitable synthon for generating more potent tetracenomycin analogs. Compared to TCMC or TCMX, 8-DMTC possesses free hydroxy groups at 4-, 8-, and 12-positions that can be modified with sugars or alkyl chains to probe structure-activity relationships.

Due to their complex mycelial life cycle, *Streptomyces* spp. fermentations are challenging to scale-up to industrial-level production. *Streptomyces* spp. tend to form mycelial aggregates, which results in poor biomass production and target product formation [24]. We envisioned four strategies to achieve the goal of high production titers of 8-DMTC: (1) deletion of the Mat complex, which contributes to hyphal aggregation [25,26]. (2) overexpression of the morphogene *ssgA*, which is responsible for peptidoglycan maintenance in actinomycetes [27]; (3) expression of the *scbr2* pseudo-gamma butyrolactone receptor, which regulates secondary metabolism in *S. coelicolor*, as well as upregulation of two genes involved in glycolysis: *gap1* (glyceraldehyde-3-phosphate dehydrogenase) and *pyk2* (pyruvate kinase) [28]. (4) our previous strategy to overexpress the acetyl-CoA carboxylase complex (e.g., *accA2BE*), which should direct more metabolic flux from acetyl-CoA to malonyl-CoA, a structural building block of 8-DMTC.

**Figure 2.**
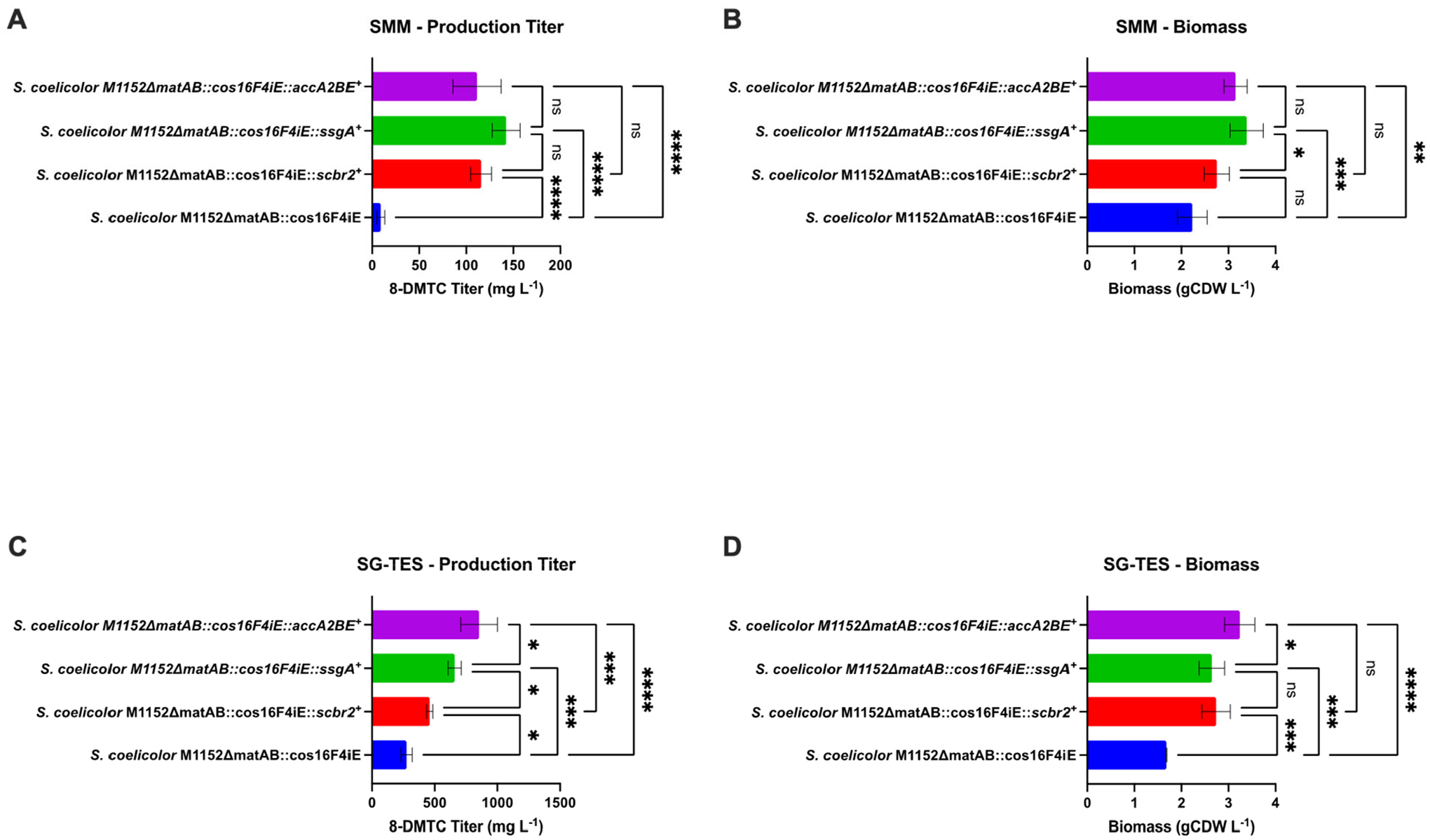
Metabolic engineering of 8-demethyl-tetracenomycin C in *Streptomyces coelicolor* M1146::cos16F4iE. (Panel (**A**)) Production titers of 8-DMTC and (Panel (**B**)) Biomass accumulation of the different strains in defined S.M.M. liquid media. (Panel (**C**)) Production titers of 8-DMTC and (Panel (**D**)) Biomass accumulation of the different strains in complex SG-TES liquid media. Experiments were conducted with 4–6 biological replicates in 50 mL shake flask cultures. Experimental groups were compared using a *t*-test to determine statistical significance (*p* < 0.05). The statistical significance of observed results was established with a *p* < 0.05. * indicates *p* ≤ 0.05, ** indicates *p* ≤ 0.01, *** indicates *p* ≤ 0.001, and **** indicates *p* ≤ 0.0001.

Previously, we developed a strain that expresses the *elm* PKS, *Streptomyces coelicolor* M1146::cos16F4iE, as a production chassis for the biosynthesis of [16] and glycosylated elloramycins [11]. First, we evaluated the impact of deleting the Mat complex on the production of 8-DMTC. Here, we expressed the cos16F4iE integration cassette, which harbors the elloramycin biosynthetic gene cluster, in the improved production chassis *Streptomyces coelicolor* M1152Δ*matAB* (Figure 2). This host produced 9 mg/L in defined S.M.M. liquid media and 275 mg/L in complex SG-TES media, which was an improvement over the previously reported *S. coelicolor* M1146::cos16F4iE host (160 mg/L in SG-TES media) (Figure 2). The reason for this increase in the production of 8-DMTC could be explained by the dispersed growth phenotype of the mutant, which results from disruption of the MatAB complex that specifies the output of poly-β−1,6-N-acetylglucosamine (PNAG) that leads to mycelial pellet formation [25,29]. As a result of the dispersed growth phenotype, oxygenation of the cells was increased, resulting in higher biomass productivity and 8-DMTC production titers.

Second, we sought to increase yields by overexpressing *ssgA* from *Streptomyces griseus*, which has been shown to suppress sporulation and result in fragmented growth of mycelia, thus resulting in faster growth kinetics and increased production of biomass and cell products [26]. Previously, van Wezel and coworkers demonstrated that overexpression of *ssgA* in *Streptomyces lividans* resulted in a two-fold increase in the production of enzymes in this host [30]. We cloned *ssgA* into the pENSV3 expression vector and transformed it into *S. coelicolor M1152*Δ*matAB*::cos16F4iE via conjugation. The overexpression of *ssgA* resulted in a 16-fold increase in the production of 8-DMTC (142 mg/L, *p* < 0.0001) in S.M.M. media and a 2.5-fold increase in SG-TES media (660 mg/L, *p* < 0.01) (Figure 2). Notably, the production titer increases correlated with biomass increases (*p* < 0.001 in S.M.M. and SG-TES media) (Figure 2). We reasoned that expressing *ssgA* increased both the growth kinetics of the strain and the expression of gene products from the *elm* pathway, augmenting the 8-DMTC titer.

Third, we overexpressed a pseudo-gamma butyrolactone (GBL) receptor *scbr2* in *S. coelicolor* M1152Δ*matAB*::cos16F4iE (Figure 2). Scbr2 is implicated in the biosynthesis of coelimycin P1; however, it does not bind gamma butyrolactones, but rather, it binds antibiotics, such as the endogenous actinorhodin and prodiginines, and exogenous angucyclines [31,32]. In the *S. coelicolor* M1152 mutant background, the *scbr2* gene was deleted as part of the *cpk* cluster [33]. Sulheim et al. developed a Genome-Scale Metabolic model (GEM) of the *S. coelicolor* M1152 parental strain [34], which predicted that *S. coelicolor* M1152 exhibits increased oxidative stress (i.e., flux through the tricarboxylic acid cycle) due to metabolic imbalance. The authors predicted that the overexpression of *scbr2* could upregulate glycolysis, thus relieving oxidative stress and improving biomass production [35]. To test this hypothesis, we overexpressed *scbr2* in *S. coelicolor* M1152Δ*matAB*::cos16F4iE, which gratifyingly resulted in a 13-fold improvement in 8-DMTC production titer (115 mg/L) in defined S.M.M. media and a 1.7-fold improvement in SG-TES media (460 mg/L) (Figure 2). These increases were statistically significant (*p* < 0.0001) and were similarly biomassdependent (e.g., n.s. in S.M.M. media, *p* < 0.001 in SG-TES media) (Figure 2). We rationalized that the expression of *scbr2* may upregulate glycolysis, which improved metabolic flux to biomass generation and 8-DMTC biosynthesis.

Fourth, we investigated the impact of overexpression of *accA2BE* in the M1152Δ*matAB* background. Previously, we overexpressed the acetyl-CoA carboxylase genes *accA2BE* in *S. coelicolor* M1146::cos16F4iE, which resulted in the highest production titer of 400 mg/L 8-DMTC [36]. We co-expressed the *accA2BE* genes in the *S. coelicolor* M1152Δ*matAB*::cos16F4iE strain to investigate the impact of both mutations on 8-DMTC production. This resulted in a 12-fold increase of 8-DMTC output in defined S.M.M. media (111 mg/L, *p* < 0.0001) and a 3-fold increase (854 mg/L, *p* < 0.0001) in SG-TES media as compared to the *S. coelicolor* M1152Δ*matAB*::cos16F4iE parent strain. This production increase was also driven by increased biomass (*p* < 0.01 in S.M.M. media and *p* < 0.0001 in SG-TES media). These results were demonstrated previously in the M1146 background [16]. Altogether, this shows the powerful combination that overexpression of *accA2BE, ssgA*, and *scbr2* could exert on increases in biomass and polyketide production in actinomycetes.

### 3.2. Cell Viability Assay

**Figure 3.**
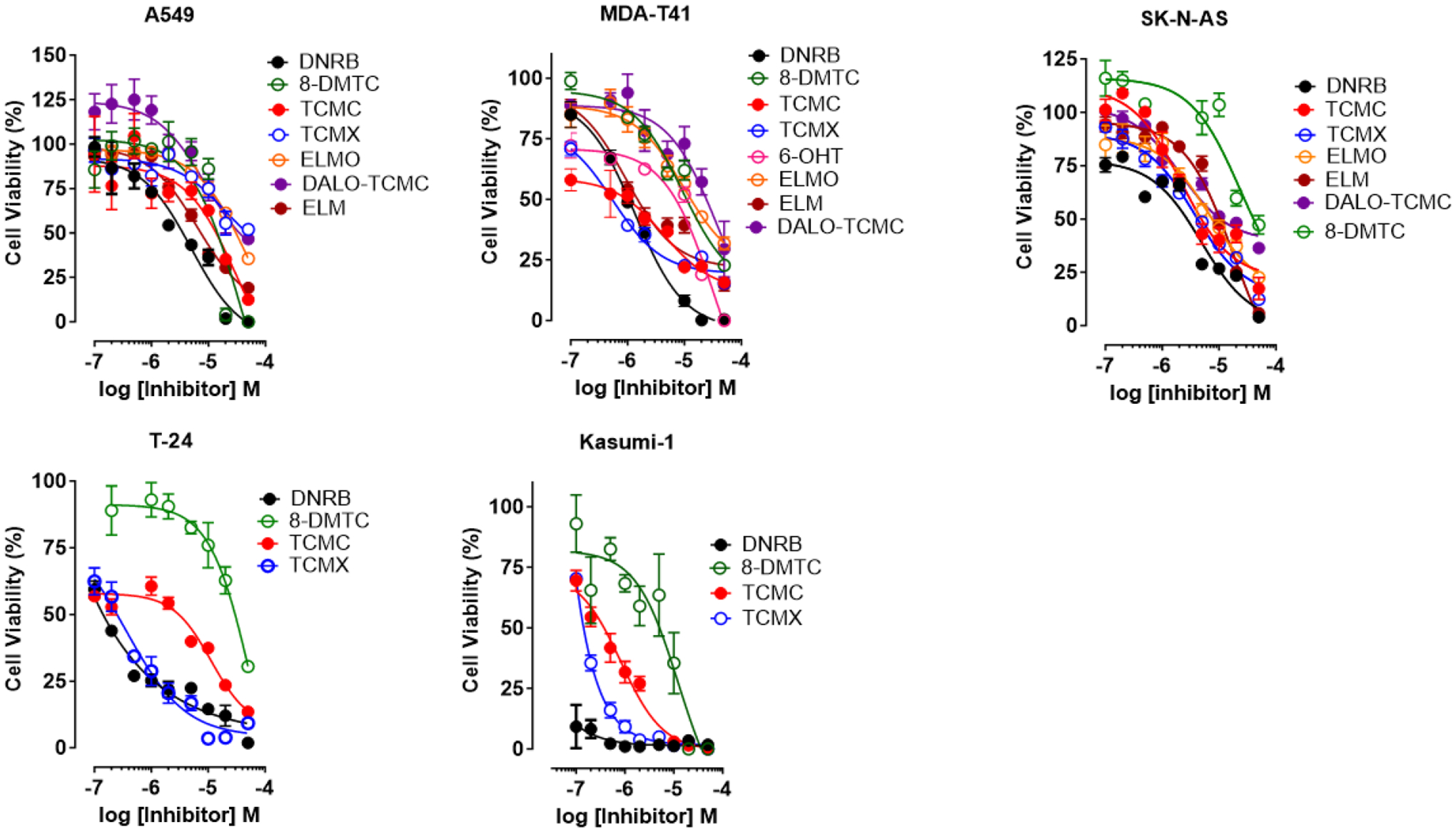
Tetracenomycins suppress viability of human lung adenocarcinoma cells (A549), thyroid cancer cells (MDA-T41), neuroblastoma cells (SK-N-AS), urinary bladder cancer cells (T-24), and acute myeloblastic leukemia cells (Kasumi-1) cells. Cultured cells seeded in 96-well plates at a density of 1 × 10^4^ were exposed to tetracenomycins (0–50 μM) for 48 h as described in the methods. Cell viability was determined after the final treatment by fluorescence using the resazurin reduction assay. Each point represents the mean ± S.E.M (n = 8). relative to the control untreated cells.

To determine the anticancer potential of tetracenomycins, we treated A549, MDA-T41, SK-N-AS, T-24, and Kasumi-1 cells with increasing concentrations of DNRB, TCMC, TCMX, and 8-DMTC (0.1–100 μM). The selected cell lines were treated with ELM, ELMO, and DALO-TCMC (0.1–100 μM). Also, the cytotoxicity of increasing concentrations (0.1–100 μM) of 6-OHT was tested on MDA-T41 cells over 48 h. The cell viability results showed that TCMX treatment decreased cell viability in a concentration-dependent manner (Figure 3). For example, in the A549 cells, treatment with TCMX, TCMC, 8-DMTC, ELM, ELMO, and DALO-TCMC showed an IC_50_ of 19.7 ± 5.3 μM, 26.4 ± 4.5 μM 37.5 ± 7.4 μM, 6.5 ± 2.2 μM, 44.6 ± 6.7 μM, and 8.8 ± 2.9 μM, respectively (Table 1). Similarly, TCMX, TCMC, 8-DMTC, ELM, ELMO, DALO-TCMC, and 6-OHT inhibited the viability of MDA-T41 cells, SK-N-AS cells, T-24 cells, and Kasumi-1 cells in a concentration-dependent manner with IC_50_s as indicated in Table 1. Furthermore, the cytotoxic effects of tetracenomycins were compared with those of daunorubicin (DNRB), which is an anthracycline cytotoxic agent that inhibits topoisomerase II and induces cellular apoptosis. Due to the unavailability of other ribosome-inhibitory agents, daunorubicin was included as a surrogate positive control due to its potency and apoptotic mechanism. The tetracenomycins were relatively less potent at reducing the viability of the selected cell lines than DNRB (Figure 3). DNRB was very efficacious and potent with IC_50_s of 5.0 ± 1.3 μM, 1.3 ± 0.3 μM, 4.9 ± 1.2 μM, 0.002 ± 0.0 and 0.04 ± 0.01 μM on A549, MDA-T41, SK-NAS, T-41, and Kasumi-1, respectively (Table 1).

Demethylation at position 8 on TCMC to yield 8-DMTC reduced the potency of TCMC, as indicated by the IC_50_ in all the cell lines. For instance, IC_50_ of 8-DMTC on SK-NAS is 23.4 ± 6.4 μM compared to 2.2 ± 0.6 μM for TCMC. However, there was an increase in efficacy in A549 cells with 100% maximal inhibition by DMTC compared to 87.5 ± 1.4% by TCMC. Although 6-hydroxylation of TCMC improved both the potency and efficacy in MDA-T41 cells, modification of TCMC with sugar moieties slightly improved the potency, as shown in ELM. However, the ketone modification of ELM to ELMO decreased the relative efficacy and potency in all the cell lines tested in SK-NAS cells. The relative potency and efficacy of DALO-TCMC are less compared to TCMC except for an increase in potency in A549 cells.

### 3.3. D Cell Viability Assay

**Figure 4.**
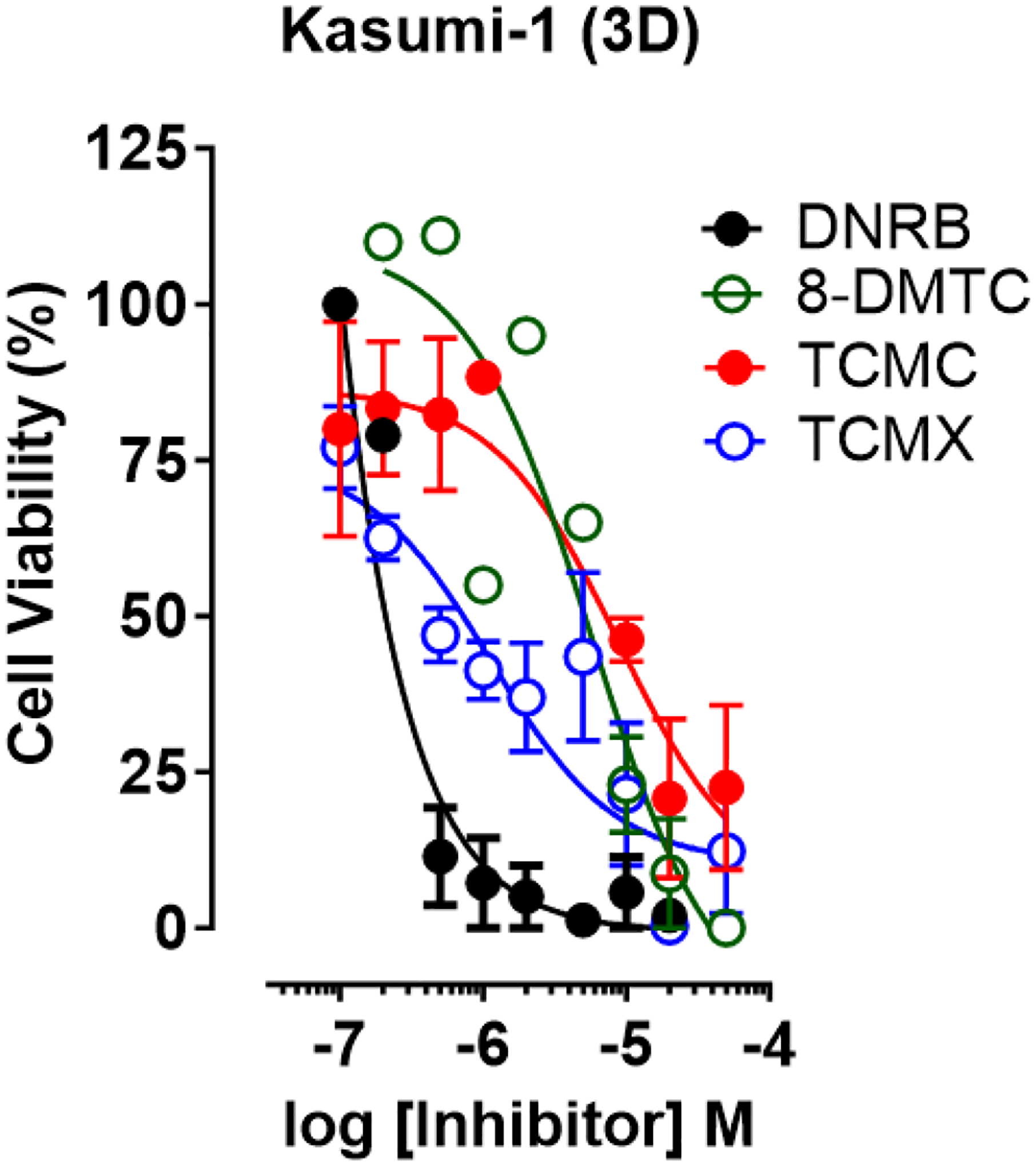
Cell viability of 3D spheroid cultures of Kasumi-1 cells was determined after the final treatment with Tetracenomycins (1–100 μM) using the CellTiter-Glo^®^ 3D assay. The EC_50_s were higher in the spheroids than in the 2D culture. Each point represents the mean ± S.E.M (n = 8). relative to the control untreated cells.

**Figure 5.**
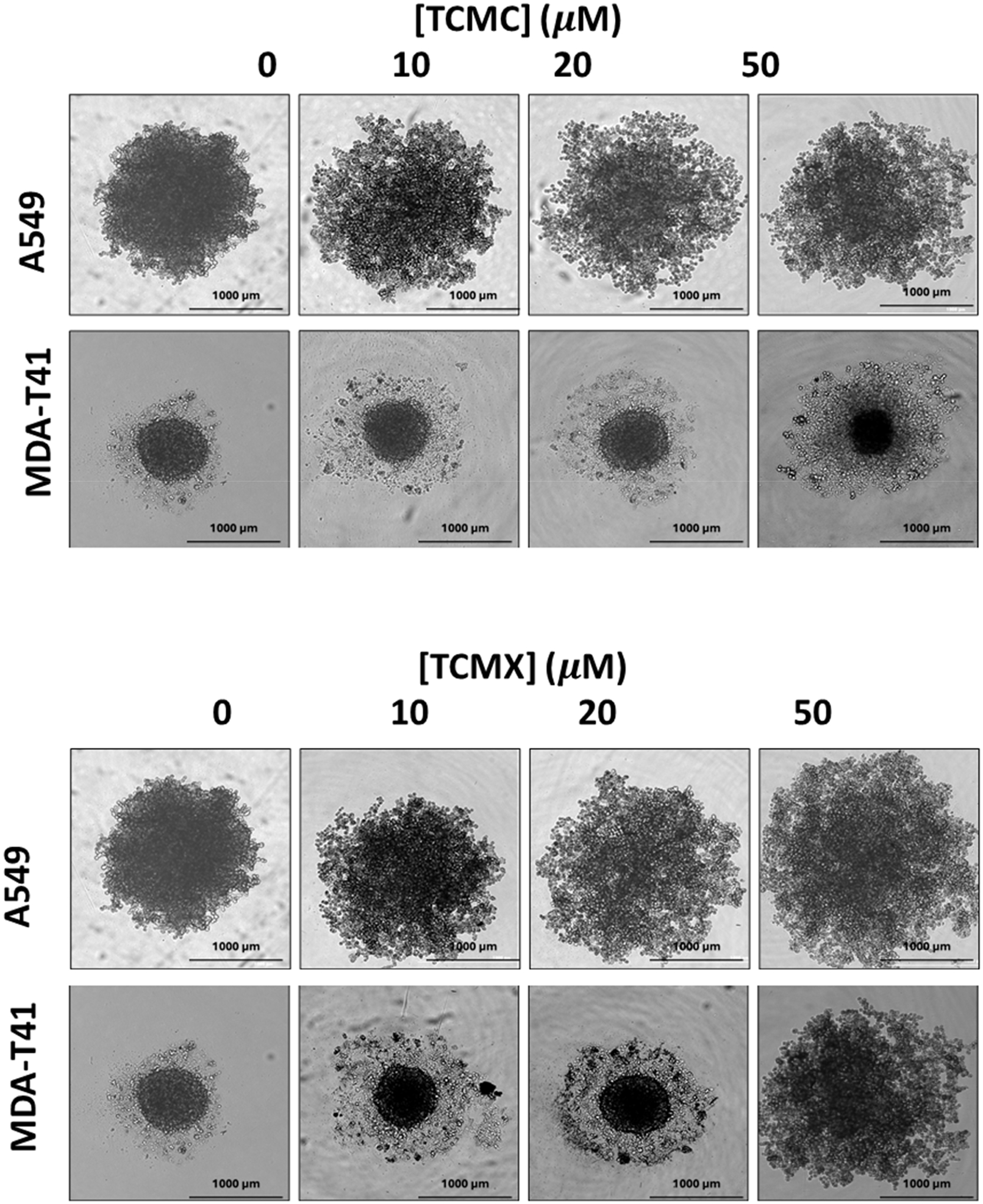
Tetracenomycins induce degeneration of 3D spheroid cultures. Representative images of 3D cultured A549 and MDA-T41 cells treated with tetracenomycin C (TCMC) and tetracenomycin X (TCMX). A549 and MDA-T41 plated at 10,000 cells/well in low-attachment, U-shaped, black, clear-bottom plates. Images were acquired 48 h after treatment. Representative images of spheroids treated with different tetracenomycins concentrations are shown. Spheroids treated with high concentrations of tetracenomycins appear to disintegrate.

The effects of TCMC and TCMX were studied using 3D spheroids culture to help observe drug response characteristics in a system that better simulates the in vivo situation of tumors [20]. Cell viability assays revealed a concentration-dependent effect of TCMX, TCMC, 8-DMTC, and DNRB (the reference compound) on A549, MDA-T41, and Kasumi-1 cell spheroids, as shown in Figure 4. TCMC and TCMX degenerated the established spheroids generated with A549, MDA-T41, or Kasumi-1 cells. The induction of spheroid degeneration and cell death occurred at relatively higher concentrations than the 2D monolayer culture of the cell lines tested, as indicated in Figure 5. Additionally, in the Kasumi-1 cells, treatment with TCMX, TCMC, 8-DMTC, and DNRB showed an IC_50_ value of 8.8 ± 2.4 μM, 1.3 ± 0.2 μM 5.7 ± 2.5 μM, and 0.004 ± 0.0 μM, respectively.

### 3.4. Colony Forming Assay

**Figure 6.**
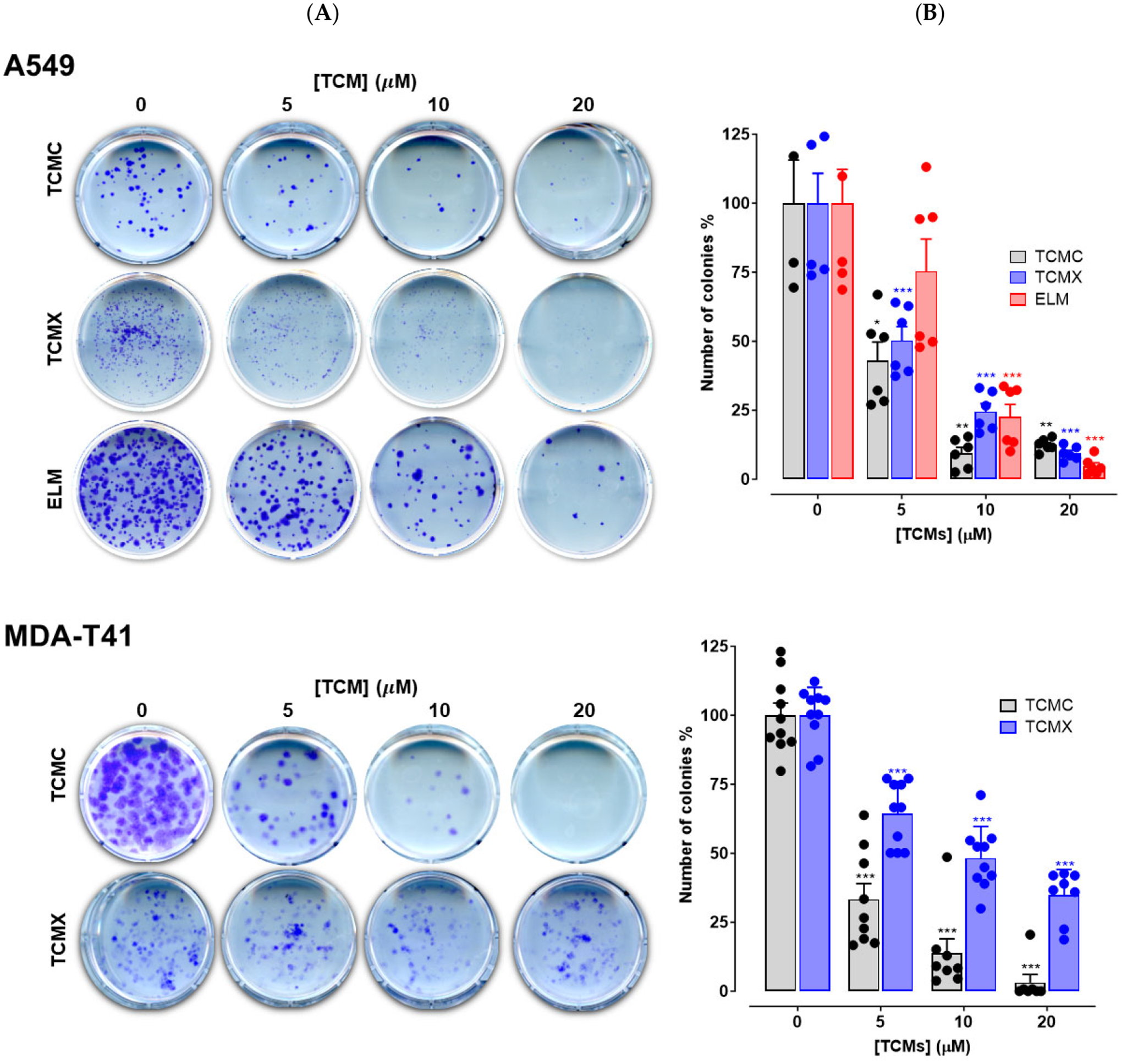
Clonogenic cell death caused by Tetracenomycins. Cultured cells were treated with TCMC, TCMX, or ELM (0–20 μM) for 72 h, washed, counted, and incubated for 10–14 days at 37 °C and clonogenic survival was determined. (**A**) Representative images of cells treated with TCMC, TCMX, or ELM are shown. (**B**) The number of colonies formed was counted, and the results are expressed as the means (± S.E.M., n = 8) relative to the controls. Significance (* *p* < 0.05; ** *p* < 0.01; *** *p* < 0.001) was determined by Student’s *t*-test.

We further explored the ability of cancer cells to survive, grow, and form colonies after exposure to tetracenomycins. Clonogenic or colony-forming assays were conducted with A549 and MDA-T41 cells to determine the ability of tetracenomycins to inhibit individual cells from forming colonies. We observed that TCMX induced concentration-dependent inhibition of cell survival and colony formation in A549 and MDA-T41 cells. Cells treated with different concentrations of tetracenomycins (0–20 μM) showed a concentration-dependent reduction in colony formation for both cell lines. As shown in Figure 6, exposure of A549 cells to 5–20 μM of TCMX, TCMC, and ELM for 72 h, followed by replating the cells and allowing them to grow for 10–14 days, reduced cancer cell survival. Although TCMC and TCMX effectively inhibited colony formation at 10 μM in A549 and MDA-T41 cells, as shown in Figure 5, TCMC was relatively more effective at inhibiting clonogenic cell survival in MDA-T41 cells. At 20 μM of TCMC, no colonies were found in the MDA-T41 cells.

### 3.5. Apoptosis Assay

**Figure 7.**
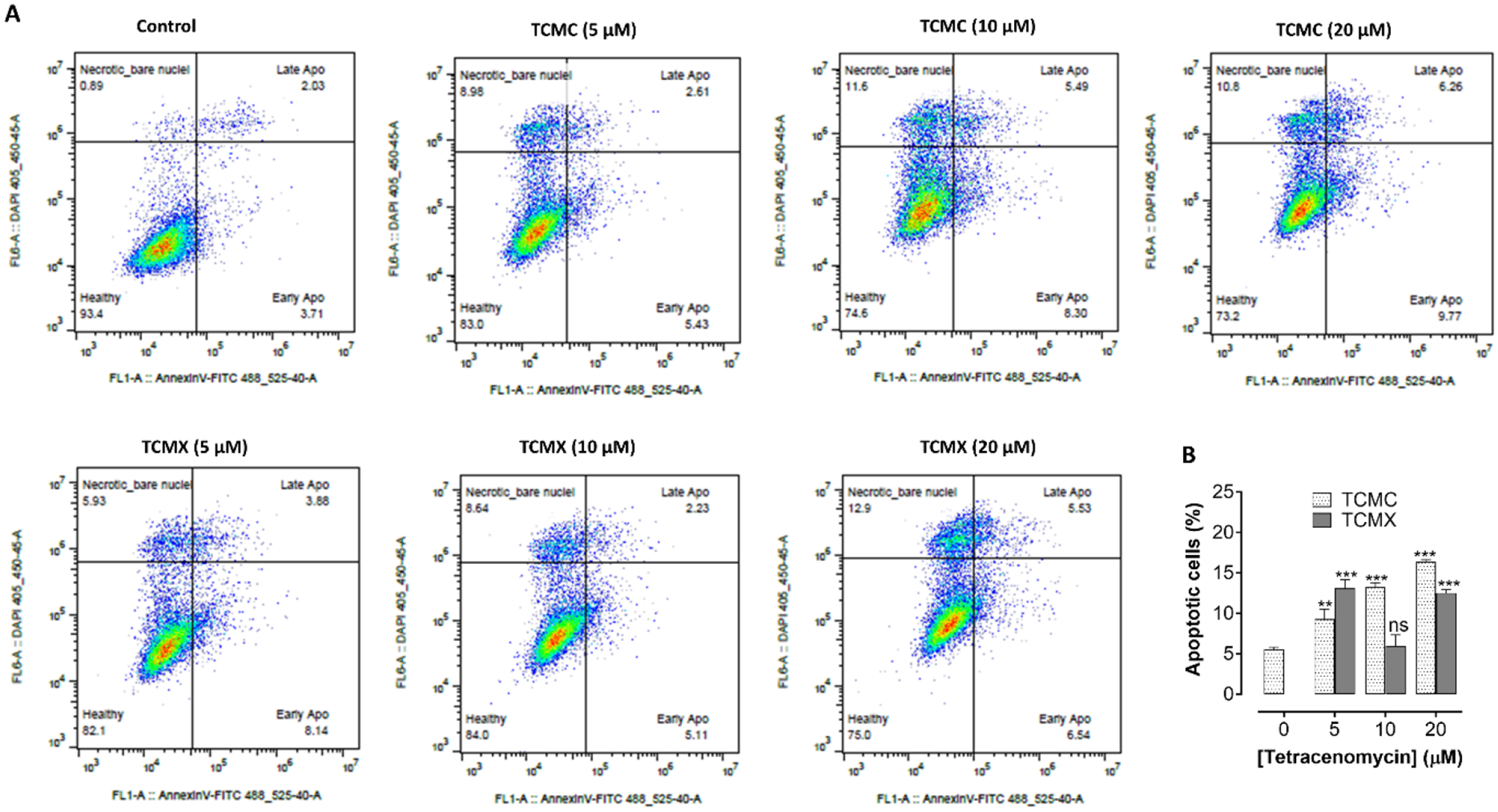
Tetracenomycins induced apoptosis in A549 lung cancer cells. (**A**) Representative scatter plots illustrating Annexin V-FITC/PI staining of cells after treatment of A549 cells with TCMC and TCMX (0–20 μM) for 72 h. Annexin-V vs. P.I. plots were generated via flow cytometry cell sorting technology. The percentage of apoptotic cell death increased in cells treated with TCMC and TCMX. (**B**) A plot of the rate of apoptotic cells (early and late apoptotic cells) after exposure to TCMC and TCMX. Data were expressed as means (±SEM, n = 3). ** *p* < 0.01; *** *p* < 0.001 indicate significant differences compared to control untreated cells.

Cytotoxicity induced by tetracenomycins may result from necrosis or apoptosis. It is desirable to induce apoptosis for an anticancer effect because it is not associated with significant inflammation. To assess the mode of cell death caused by tetracenomycins, we conducted flow cytometry analysis on A549 cells exposed to increasing concentrations of the tetracenomycins using the Annexin V/propidium iodide test for apoptosis. Figure 7A,B show a significant cell increase with an increased Annexin V-FITC fluorescence in samples treated with higher concentrations of tetracenomycins relative to the vehicle-treated control group (Figure 7). We evaluated the concentration-dependent effects on the early/late apoptotic population of A549 cells when treated with 5.0, 10 or 20 μM of TCMC (9.3 ± 0.7%, 13.2 ± 0.3%,16.4 ± 0.2%) and TCMX (13.1 ± 0.6%, 6.4 ± 0.9%, 12.5 ± 0.2%), respectively, compared to the control (5.6 ± 0.3%) (Figure 7). Substantial necrosis was also observed in the A549 cells exposed to both compounds. Although we did not observe a very robust level of apoptosis, a further analysis of total cell counts after exposure to the compounds, indicated that the tetracenomycins significantly inhibited cell growth, accounting for the significant decrease in cell viability observed earlier.

## 4. Discussion

Tetracenomycins, from the polyketide family of natural products, exhibit significant antibacterial effects and antitumor activity [3]. Some studies have reported that tetracenomycins possess antitumor activity in several in vitro and in vivo models [21,37,38]. Qiao et al. recently showed that tetracenomycin X selectively attenuates the proliferation of lung cancer cells through the induction of cell cycle arrest, which was the primary antitumor mechanism in lung cancer cells [20]. Osterman et al. demonstrated that the mechanism of action for the tetracenomycins is the inhibition of translation by binding to the nascent peptide exit tunnel of the mammalian 60S ribosome subunit and the *Escherichia coli* 50S ribosome subunit [9]. Tetracenomycins stack on top of the noncanonical base pairs U1782 and U2586 of the 23S ribosomal RNA. Leroy et al. shed further light on this mechanism of action by demonstrating that tetracenomycin X primarily inhibits peptide bond formation between incoming aminoacyl-tRNA and QK motifs in the nascent polypeptide [10]. Tetracenomycin C, tetracenomycin X, and elloramycin are analogs that feature either a methyl group at 8-position or a permethylated L-rhamnose sugar, which increases their binding within the hydrophobic pocket and antitumoral activity.

Here, we further improved on our metabolic engineering platform for the increased production of valuable intermediate 8-DMTC. We implemented precursor substrate engineering (*accA2BE*) with global regulatory (*scbR2*) and morphology (*ssgA*) engineering to enhance product titers. We previously demonstrated this approach to enhance the production of anthracyclinones [39,40]. Here, these engineering approaches were found to be additive to the impact of the *matAB* mutation, which gives a more dispersed growth phenotype and contributes to increased production. To the best of our knowledge, the titer of 8-DMTC reported here of 854 mg/L is the highest achieved in shake flask fermentations to date.

It is tempting to think that 8-DMTC could serve as a semi-synthetic starting material for the generation of next-generation tetracenomycins with rational modifications to the hydroxyl groups at the 8-position and 12-positions. These modifications could include acyl or alkyl chains to increase the ClogP of the drug or enhance its binding to the NPET of the mammalian 60S ribosome. Leroy et al. also showed that the TCMX 8-position methyl group interacts with U1591 in the human 60S ribosome, which would necessitate shorter alkyl chains to avoid steric clash with U1591 [10]. On the other hand, the *E. coli* ribosome TCMX binding pocket has a channel spanning the backbone phosphate groups of 23S rRNA residues G2582 and G2608. This channel could accommodate tetracenomycins with longer alkyl chains, which might provide one means for dissecting the antitumoral activity away from new tetracenomycin antibiotics.

## 5. Conclusions

In this study, we tested tetracenomycins generated from combinatorial biosynthesis efforts for their anticancer activities on cancer cells for viability and survival, and we report that the tetracenomycins inhibit the viability of a panel of cancer cell lines and suppress survival of lung cancer cells in both 2D and 3D cultures. The cytotoxic activity of tetracenomycins has been reported on various mammalian cancer cell lines. It has been associated with the compounds binding to the large ribosomal subunit and inhibiting peptide translation. Our results indicate that all the compounds tested effectively induced cancer cell death at low micromolar concentrations on all the cancer human cell lines used. TCMX and TCMC are the most potent tetracenomycins and, therefore, the most effective at inhibiting mammalian cancer growth. Further evaluation using 3D cell culture to determine the ability of the compounds to penetrate a simulated tumor shows that the compounds induced degeneration of tumor spheroids. Colony-forming assays were used to assess the ability of single cells to grow into colonies after treatment indicated that the compounds were effective at preventing tumor re-growth in vitro. Further analysis of the mode of cell death induced by TCMX and TCMC showed less robust induction of apoptosis, indicating relatively more antiproliferative action than cytotoxic effect. One of the major limitations of this study is that the assays conducted upheld the well-characterized the antiproliferative effect of the tetracenomycins, but more sensitive assays will need to be conducted to incisively untangle their pharmacodynamic mechanism. Overall, these results align with the previously reported anticancer properties of tetracenomycins, which suggested that the induction of cell cycle arrest was the central antitumor mechanism [21]. Our findings lend more credence to the anticancer potential of tetracenomycins, which can be further developed for chemotherapy.

## Supplementary Material

Supplementary Material

**Supplementary Materials:** The following supporting information can be downloaded at: https://www.mdpi.com/article/10.3390/app152211985/s1, Supplementary File S1. Table of expression constructs, table of bacterial strains, HPLC-MS UV/vis data and mass spectra for compounds used in the study. Supplementary File S2. Detailed methods used for producing and isolating tetracenomycins. References [11–22,26,40–42] are cited in the supplementary materials.

## Figures and Tables

**Table 1.** The percentage maximum inhibition and the IC_50_ of tetracenomycins on A549, MDA-T41, SK-NAS, T41, and Kasumi-1 human cancer cells.

Compounds	A549		MDA-T41		SK-NAS		T-41		Kasumi-1	
	% Max. Inhibition	IC_50_ μM	% Max. Inhibition	IC_50_ μM	% Max. Inhibition	IC_50_ μM	% Max. Inhibition	IC_50_ μM	% Max. Inhibition	IC_50_ μM
DNRB	100.0 ± 0.0	5.0 ± 1.3	99.5 ± 0.3	1.3 ± 0.3	96.0 ± 2.5	4.9 ± 1.2	98.2 ± 0.8	0.002 ± 0.0	98.2 ± 0.8	0.04 ± 0.01
TCMC	87.5 ± 1.4	26.4 ± 4.5	84.3 ± 1.6	4.3 ± 2.2	82.6 ± 5.1	2.2 ± 0.6	86.5 ± 1.3	12.3 ± 2.5	100.0 ± 0.0	0.82 ± 0.1
TCMX	48.0 ± 2.9	19.7 ±5.3	84.6 ± 1.5	0.54±0.3	87.6 ± 0.8	3.7 ± 1.8	90.7 ± 1.2	0.36 ± 0.4	100.0 ± 0.0	0.09 ± 0.0
8-DMTC	100.0 ± 0.0	37.5 ± 7.4	77.2 ± 0.7	9.2 ± 3.1	52.8 ± 4.3	23.4 ± 6.4	69.3 ± 2.0	60.4 ± 7.5	100.0 ± 0.0	12.5 ± 2.1
6-OHT	-	-	100.0 ± 0.0	3.4 ± 1.3	-	-	-	-	-	-
ELMO	64.4 ± 2.2	44.6 ± 6.7	68.2 ± 2.5	9.7 ± 3.6	77.6 ± 2.7	6.6 ± 2.4	-	-	-	-
ELM	81.0 ± 2.6	6.5 ± 2.2	85.4 ± 2.5	1.0 ± 0.5	94.2 ± 1.8	18.3 ± 6.2	-	-	-	-
DALO-TCMC	53.4 ± 2.9	8.8 ± 2.9	70.5 ± 11.5	37.9 ± 4.8	63.6 ± 1.5	2.1 ± 0.5	-	-	-	-

## Data Availability

The original contributions presented in this study are included in the article/Supplementary Material. Further inquiries can be directed to the corresponding author.
