# Supplementary material for: Tetracenomycin Aglycones Primarily Inhibit Cell Growth and Proliferation in Mammalian Cancer Cell Lines
Source: Appl Sci (Basel). Author manuscript; Available in PMC 2026 Jun 16. (PMC13267936; doi:10.3390/app152211985)
Supplement: Supplementary Material [file NIHMS2183971-supplement-Supplementary_Material.zip › Supplementary File S1.pdf]

Table of Contents

Table S1. Plasmids used in this study.. 2

Table S2. Bacterial strains used in this study. 3

Figure S1. HPLC-UV/VIS chromatogram of 8-demethyl-tetracenomycin C standard at 254 nm (upper trace) and 411 nm (lower trace). 4

Figure S2. UV-vis spectrum of 8-demethyl-tetracenomycin C.. 5

Figure S3. ESI-MS -ve mode mass spectrum of 8-demethyl-tetracenomycin C. 6

Figure S4 HPLC-UV/vis chromatogram of tetracenomycin C standard at 254 nm (upper trace) and 411 nm (lower trace). 7

Figure S5. UV-vis spectrum of tetracenomycin C. 8

Figure S6. ESI-MS -ve mode mass spectrum of tetracenomycin C. 9

Figure S7. HPLC-UV/vis chromatogram of tetracenomycin X standard at 254 nm (upper trace) and 411 nm (lower trace). 10

Figure S8. UV-vis spectrum of tetracenomycin X standard. 11

Figure S9. ESI-MS -ve mode mass spectrum of tetracenomycin X standard. 12

Figure S10. HPLC-UV/vis chromatogram of elloramycinone standard at 254 nm (upper trace) and 411 nm (lower trace). 13

Figure S12. ESI-MS -ve mode mass spectrum of elloramycinone standard............................. 15

Figure S13. HPLC-UV/vis chromatogram of 6-hydroxy-tetracenomycin C standard at 254 nm (upper trace) and 411 nm (lower trace). 16

Figure S14. UV-vis spectrum of 6-hydroxy-tetracenomycin C standard................................. 17

Figure S16. HPLC-UV/vis chromatogram of elloramycin at 254 nm (upper trace) and 411 nm (lower trace).. 19

Figure S17. UV-vis spectrum of elloramycin. 20

Figure S18. ESI-MS -ve mode mass spectrum of elloramycin standard. 21

Figure S19: HPLC-UV/vis analysis of 8-demethyl-8-O-β-D-allosyl-TCMC................................. 22

Figure S20. HPLC-MS analysis of 8-demethyl-8-O-β-D-allosyl-TCMC..................................... 23

Table S1. Plasmids used in this study.

Plasmid	Genotype and relevant characteristics	Reference
pOSV808	BioBricks®-compatible vector; *hph*^{R}, *oriT*, VWBint, *attP*, *amilCFP*	[13]
pOSV808-*accA2BE*	*ermE**p-*accA2BE* fragment cloned into pOSV808	[5]
pOSV808-*scbr2*	*scbr2* gene from *S. coelicolor* A3(2) under the control of its own promoter cloned into pOSV808	This study.
pOSV808-*ssgA*	*ssgA* gene from *Streptomyces griseus* under the control of synthetic promoter ScoSPL20 and the strong *tuf1* RBS.	This study, [14]

Table S2. Bacterial strains used in this study.

Strain	Genotype and relevant characteristics	Reference
E. coli DH5α	*fhuA2 lac(del)U169 phoA glnV44 Φ80' lacZ(del)M15 gyrA96 recA1 relA1 endA1 thi-1 hsdR17*	New England Biolabs
E. coli ET12567/pUZ8002	*F- dam*13::Tn9 *dcm*6 *hsdM hsdR zjj*-202::Tn10 *recF*143 *galK2 galT22 ara*-14 *lac*Y1 *xyl*-5 *leuB6 thi*-1 *tonA*31 *rpsL*136 *his*G4 *tsx*-78 *mtl*-1 *glnV*44; *tra, neoR, RP4*; *E. coli*-*Streptomyces* intergeneric conjugation host	[15]
S. coelicolor M1152Δ*matAB*	SCP1- SCP2- Δ*act* Δ*red* Δ*cpk* Δ*cda rpoB*(C1298T) Δ*sco2961–2962*	[16]
S. coelicolor M1152Δ*matAB*::cos16F4iE	*S. coelicolor* M1152Δ*matAB* lysogenized with cos16F4iE. Produces 8-DMTC.	This study
S. coelicolor M1152Δ*matAB*::cos16F4iE::*scbr2*	*S. coelicolor* M1152Δ*matAB*::cos16F4iE also lysogenized with pOSV808-*scbr2*	This study
S. coelicolor M1152Δ*matAB*::cos16F4iE::*ssgA*	*S. coelicolor* M1152ΔmatAB::cos16F4iE also lysogenized with pOSV808-*ssgA*	This study
S. coelicolor M1152Δ*matAB*::cos16F4iE::*accA2BE*	*S. coelicolor* M1152ΔmatAB::cos16F4iE also lysogenized with pOSV808-*accA2BE*	This study

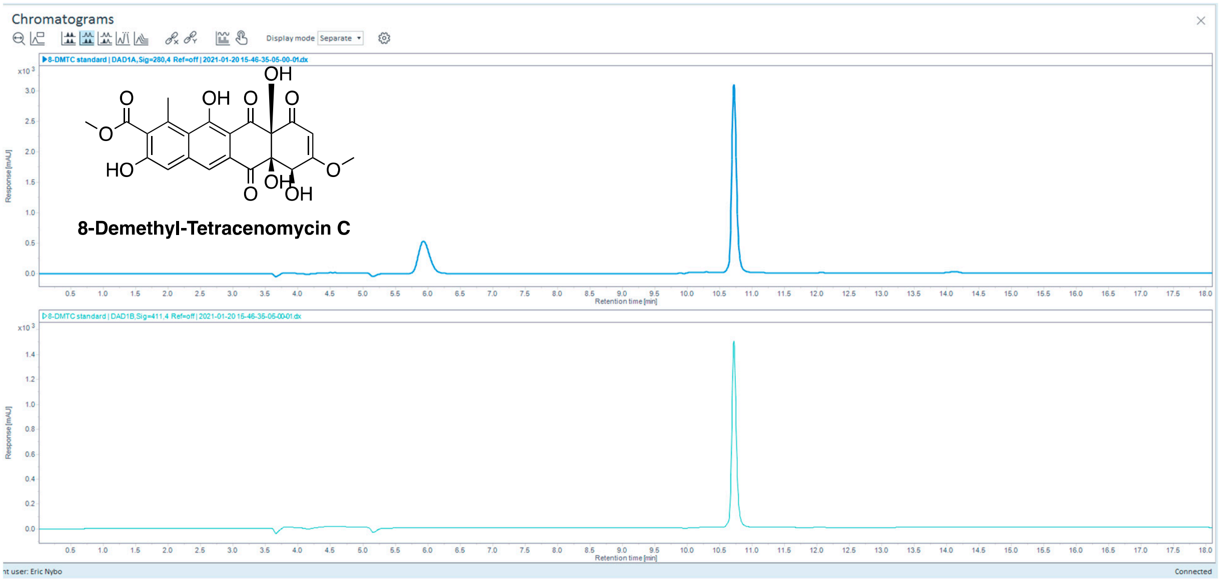

Figure S1. HPLC-UV/VIS chromatogram of 8-demethyl-tetracenomycin C standard at 254 nm (upper trace) and 411 nm (lower trace).

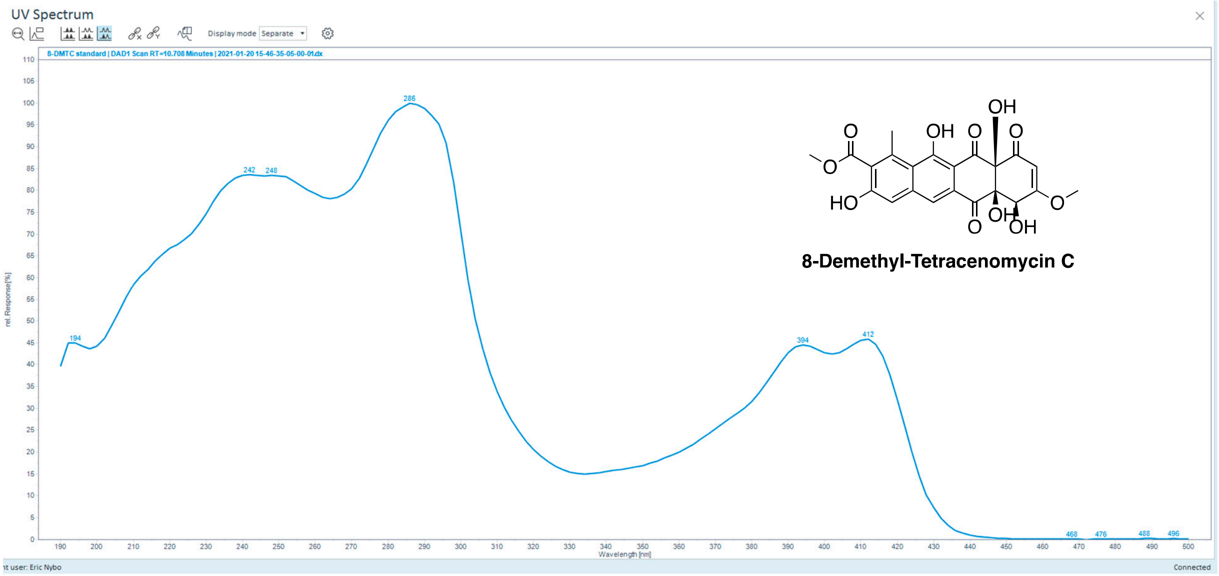

Figure S2. UV-vis spectrum of 8-demethyl-tetracenomycin C.

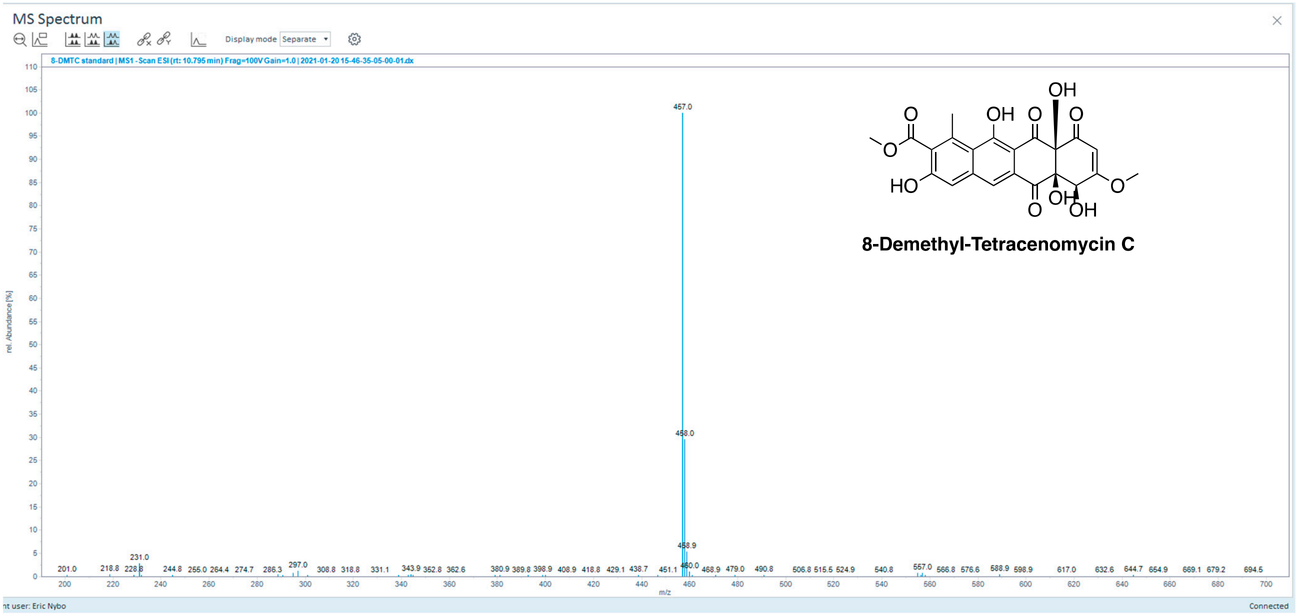

Figure S3. ESI-MS -ve mode mass spectrum of 8-demethyl-tetracenomycin C.

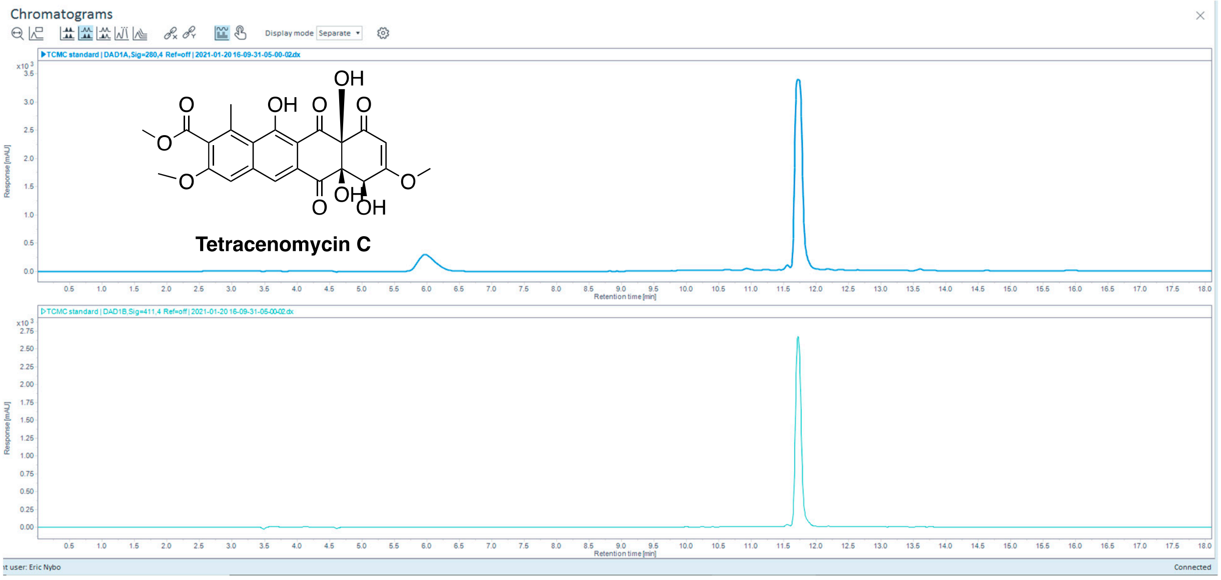

Figure S4 HPLC-UV/vis chromatogram of tetracenomycin C standard at 254 nm (upper trace) and 411 nm (lower trace).

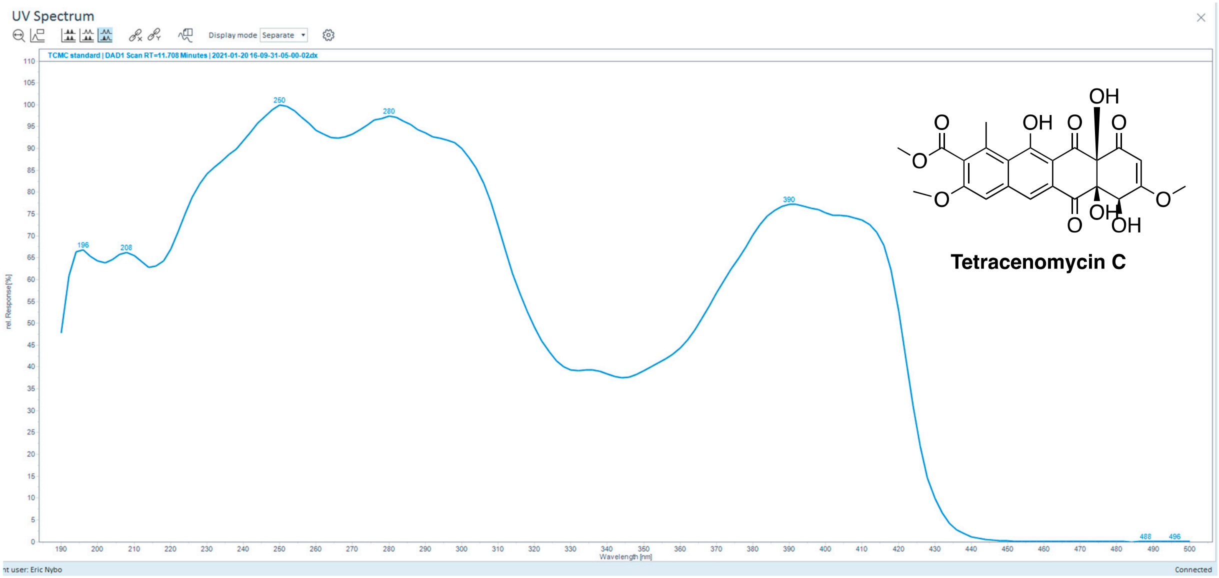

Figure S5. UV-vis spectrum of tetracenomycin C.

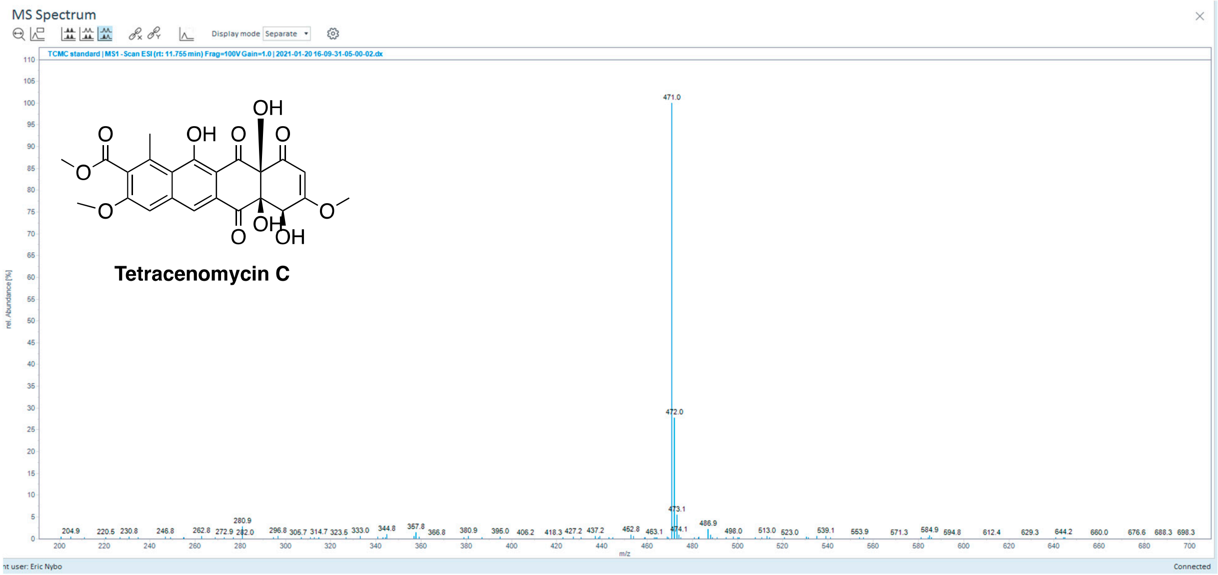

Figure S6. ESI-MS -ve mode mass spectrum of tetracenomycin C.

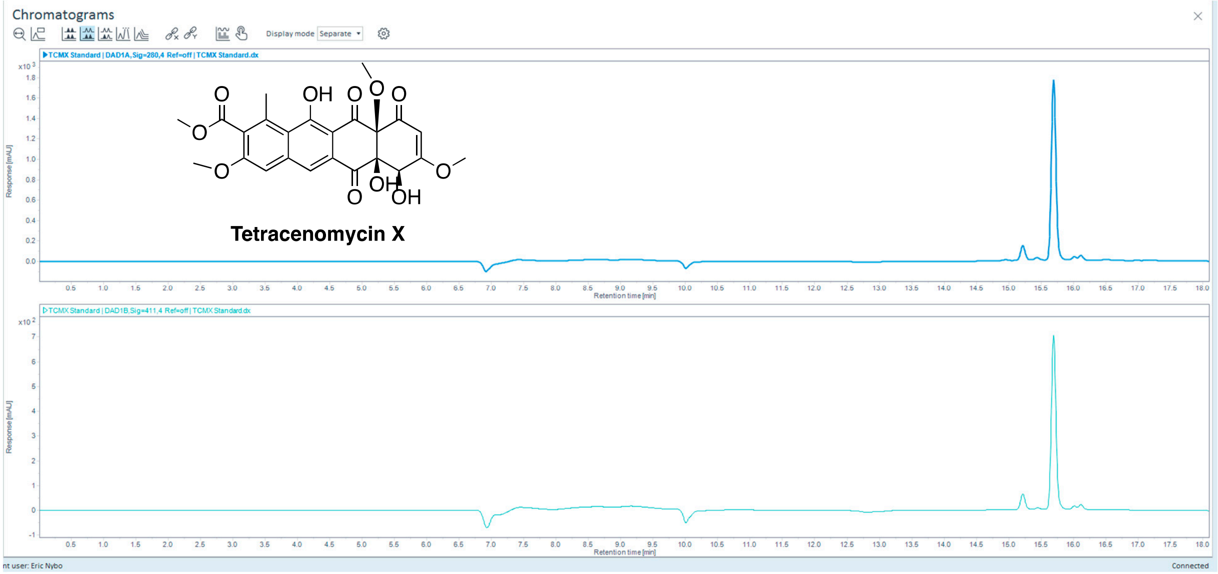

Figure S7. HPLC-UV/vis chromatogram of tetracenomycin X standard at 254 nm (upper trace) and 411 nm (lower trace).

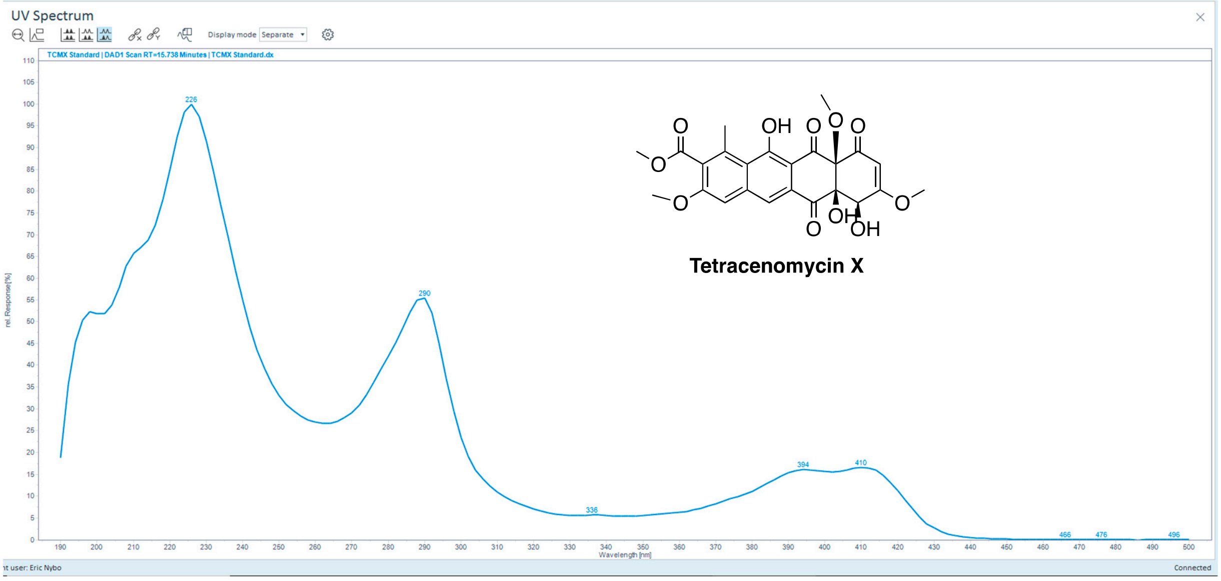

Figure S8. UV-vis spectrum of tetracenomycin X standard.

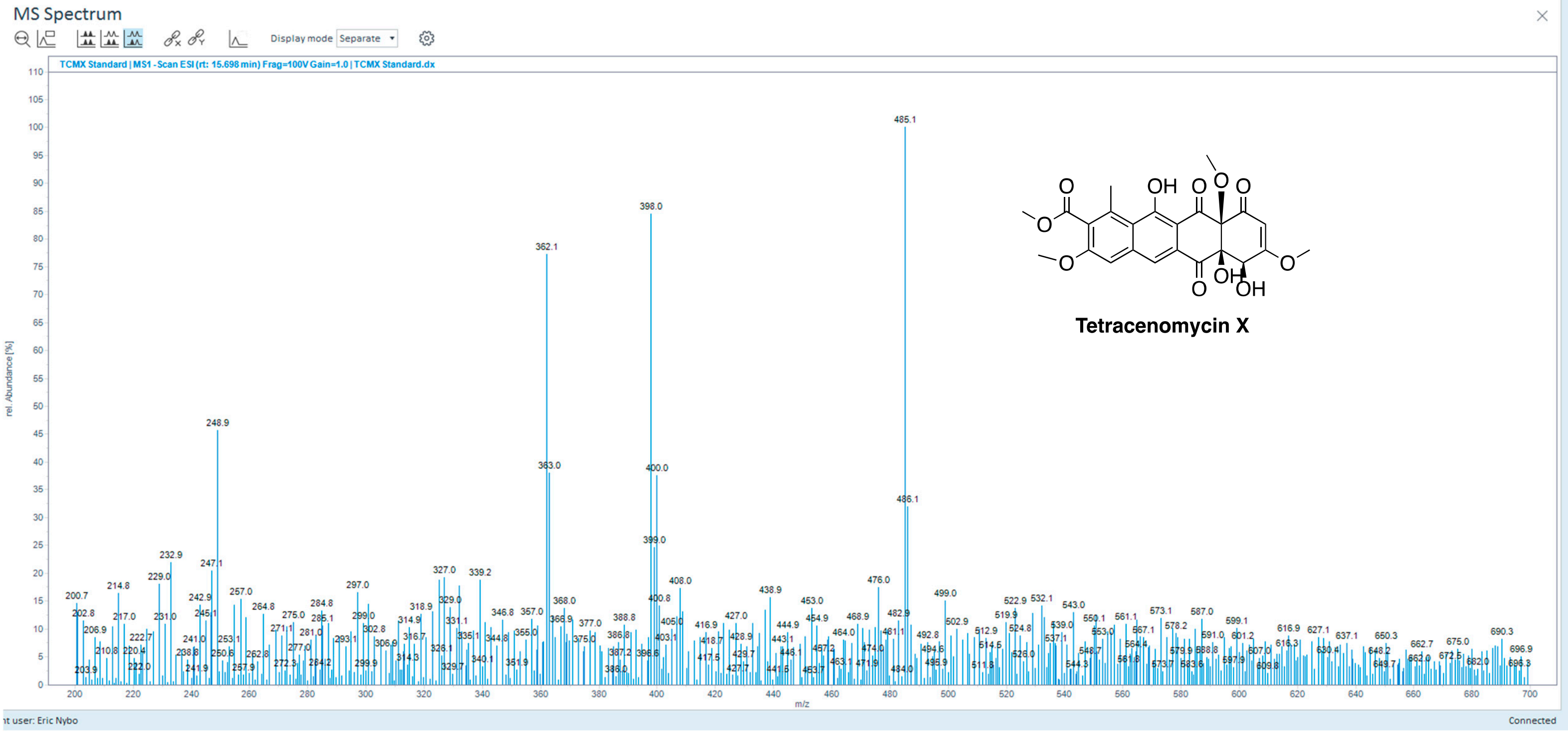

Figure S9. ESI-MS -ve mode mass spectrum of tetracenomycin X standard.

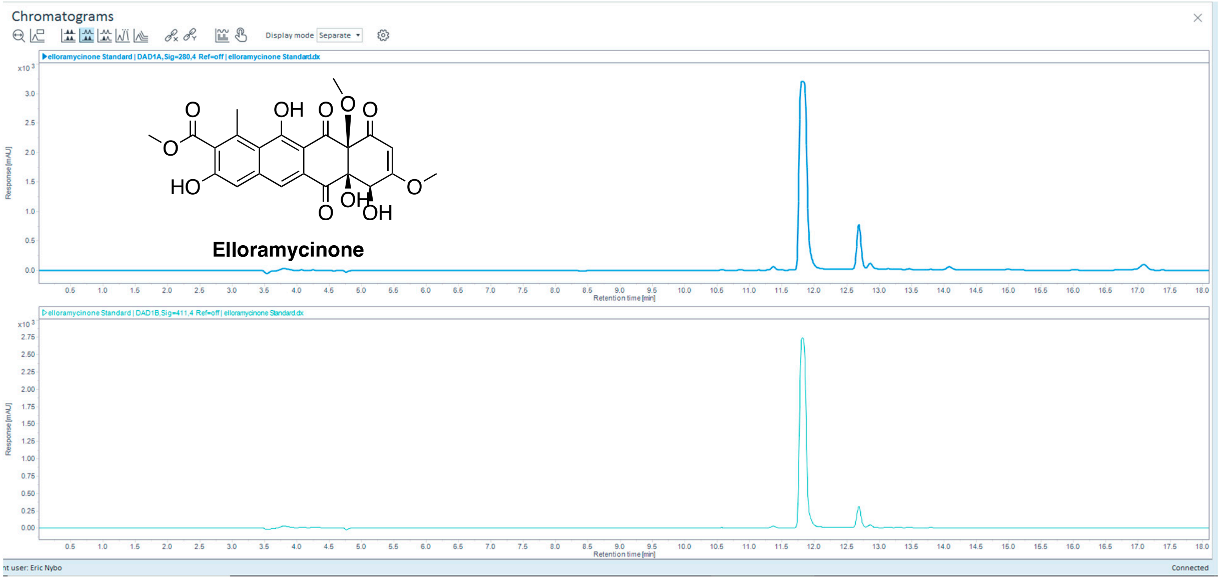

Figure S10. HPLC-UV/vis chromatogram of elloramycinone standard at 254 nm (upper trace) and 411 nm (lower trace).

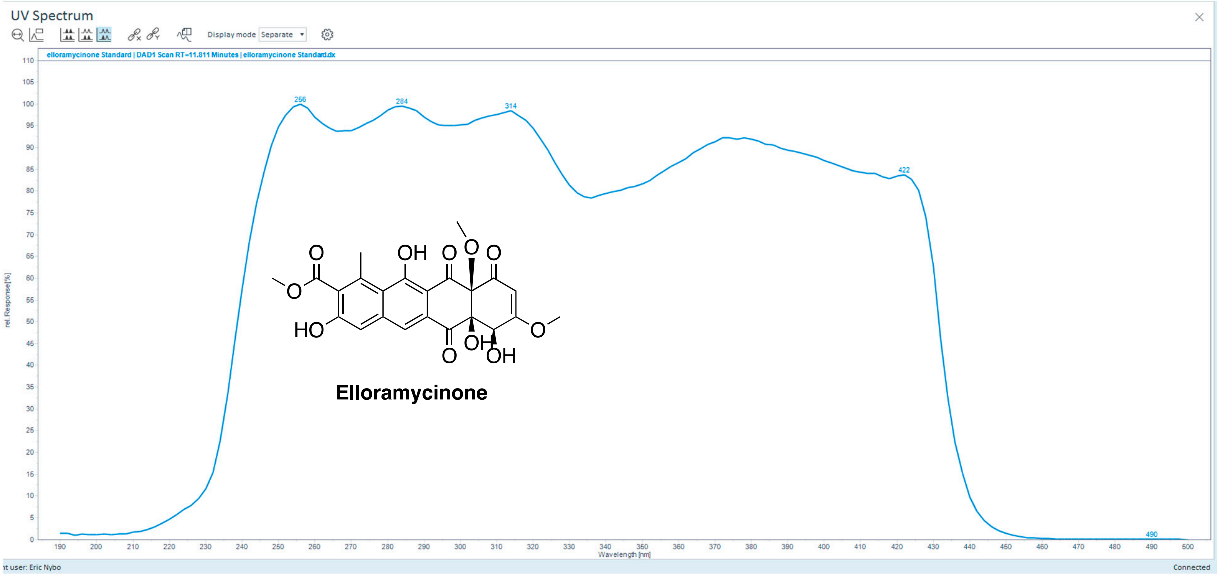

Figure S11. UV-vis spectrum of elloramycinone standard.

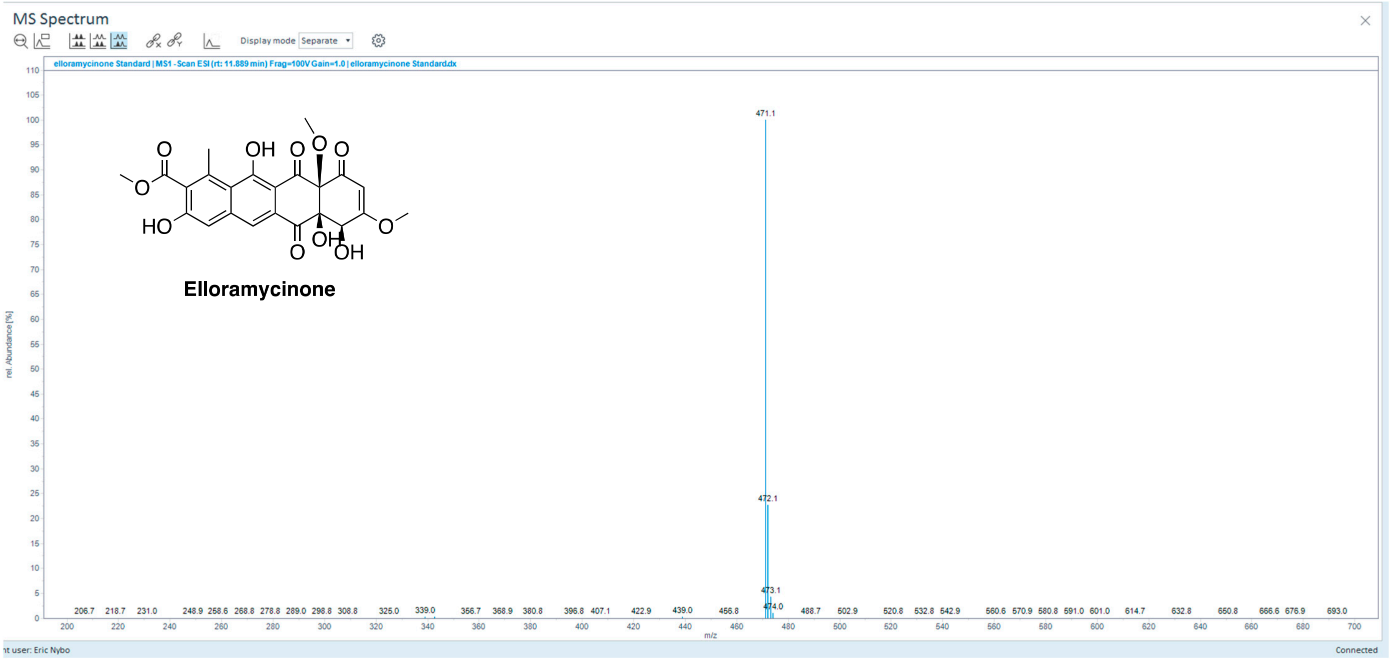

Figure S12. ESI-MS -ve mode mass spectrum of elloramycinone standard.

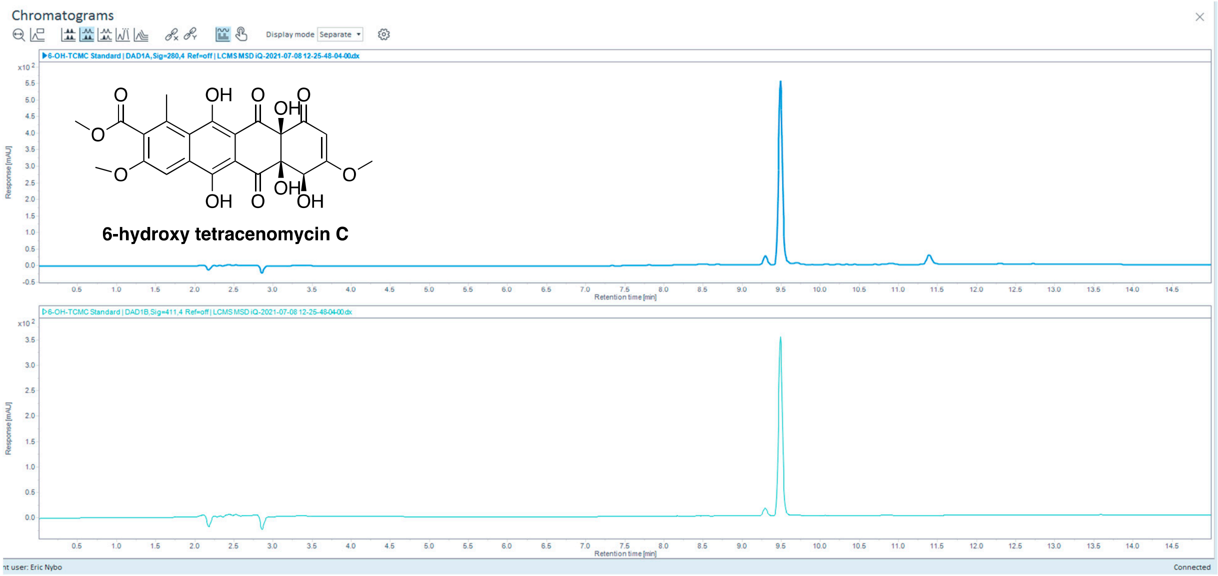

Figure S13. HPLC-UV/vis chromatogram of 6-hydroxy-tetracenomycin C standard at 254 nm (upper trace) and 411 nm (lower trace).

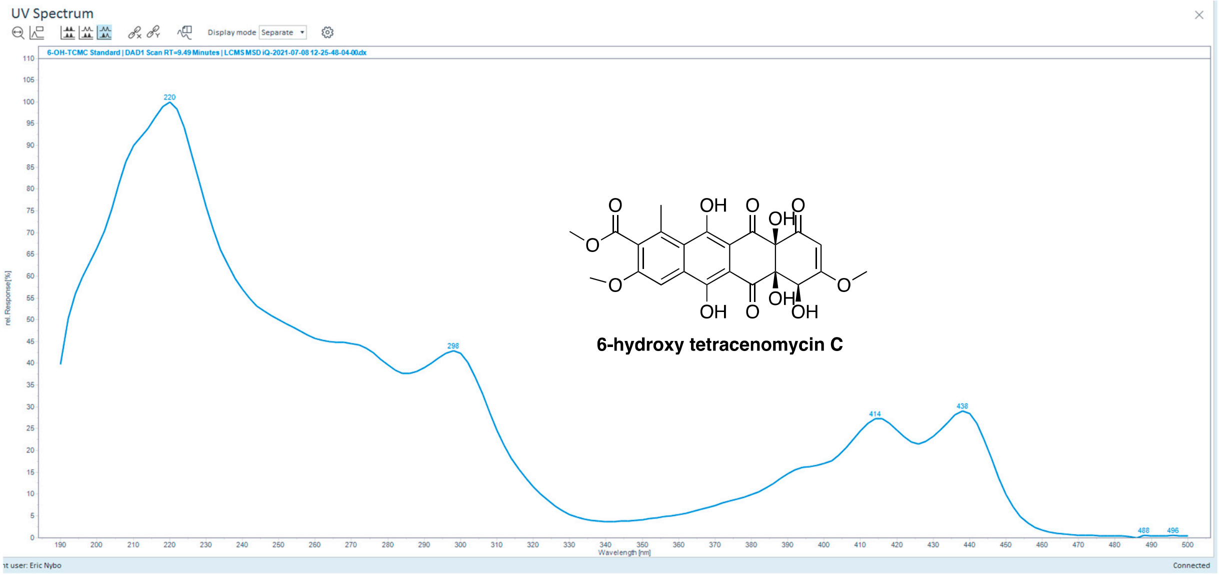

Figure S14. UV-vis spectrum of 6-hydroxy-tetracenomycin C standard.

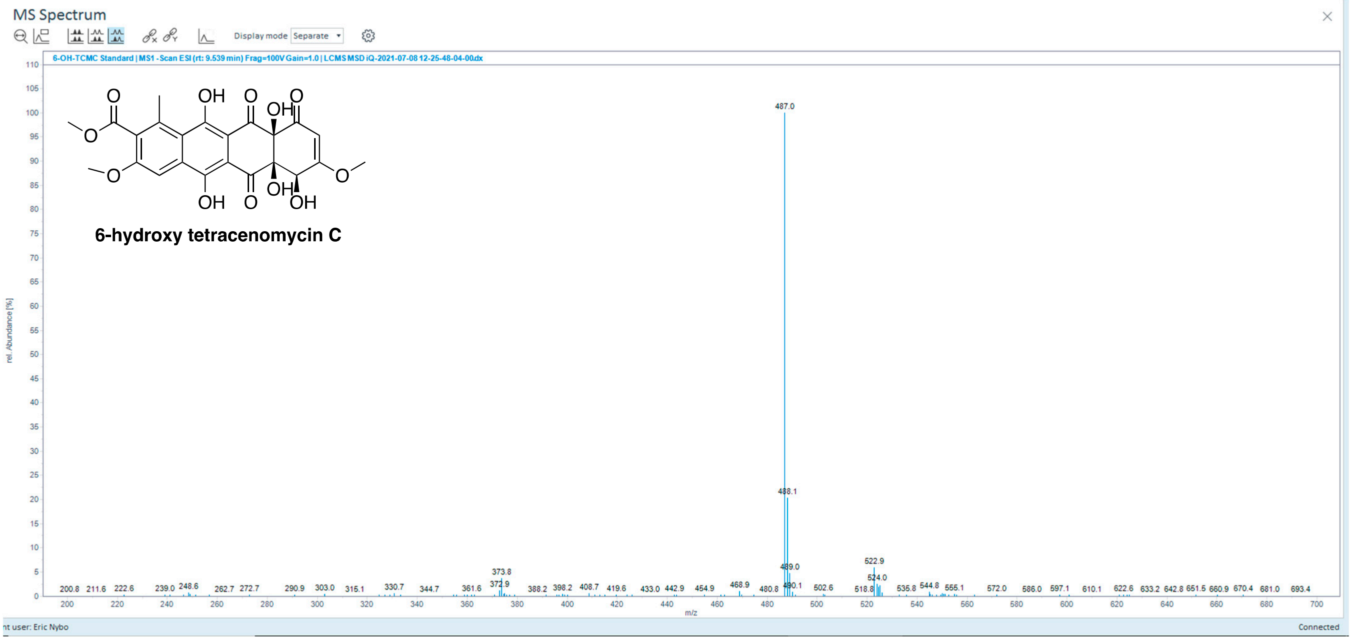

Figure S15. ESI-MS -ve mode mass spectrum of 6-hydroxy-tetracenomycin C standard.

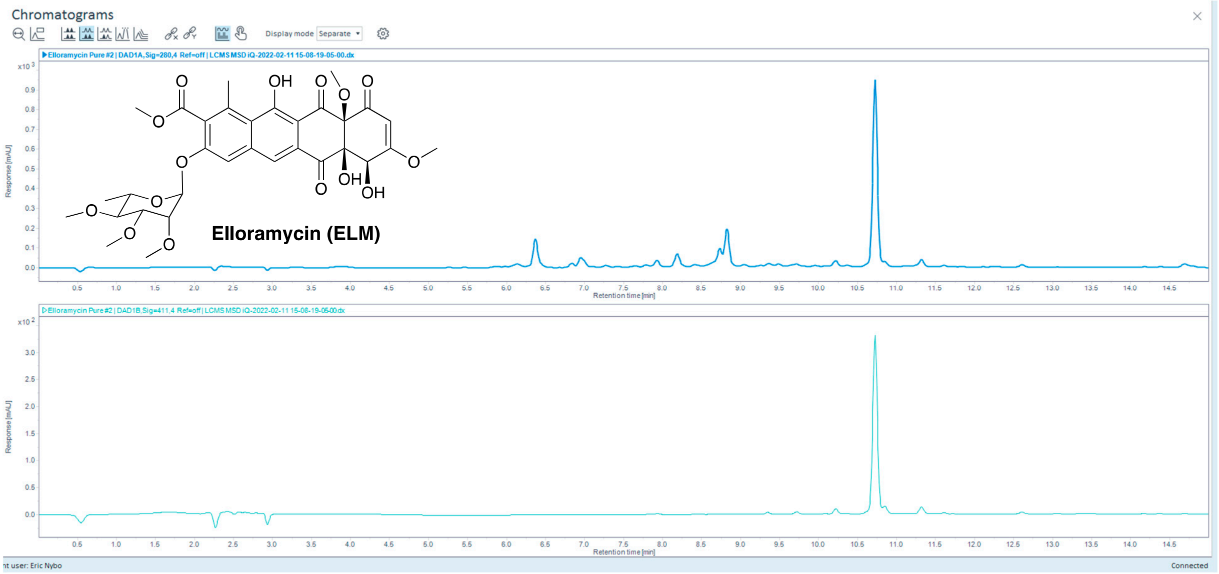

Figure S16. HPLC-UV/vis chromatogram of elloramycin at 254 nm (upper trace) and 411 nm (lower trace).

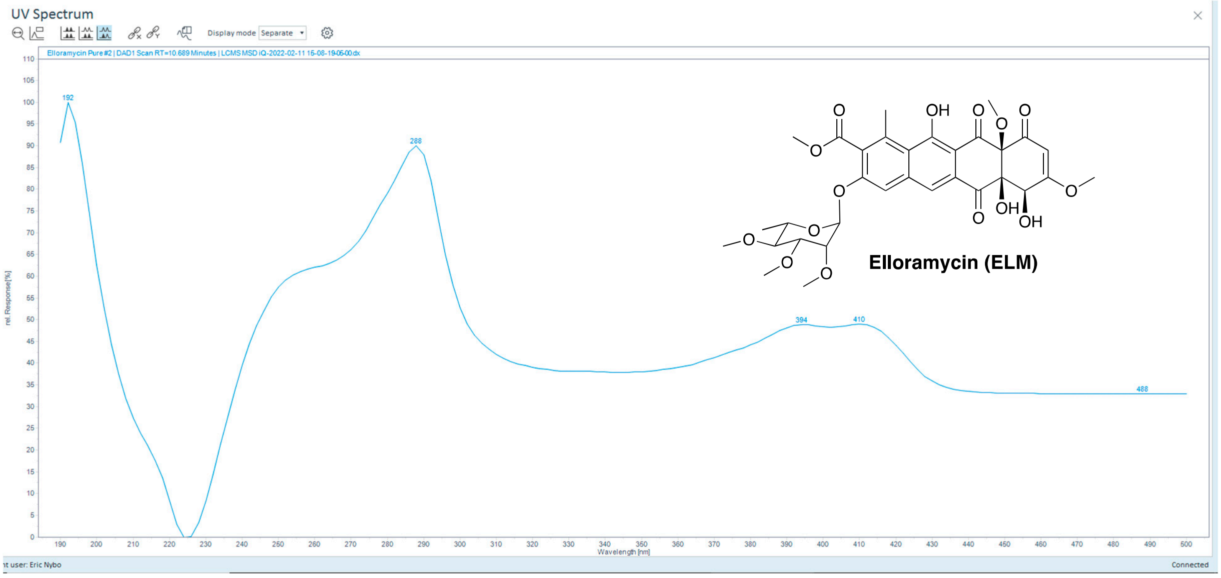

Figure S17. UV-vis spectrum of elloramycin.

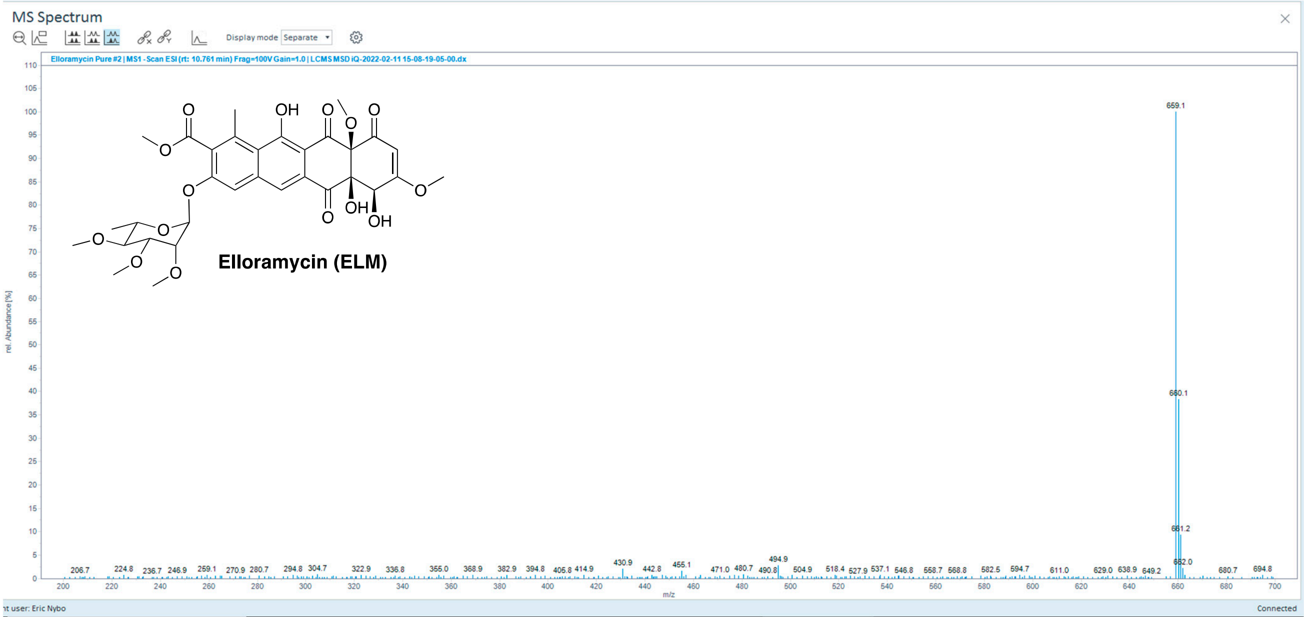

Figure S18. ESI-MS -ve mode mass spectrum of elloramycin standard.

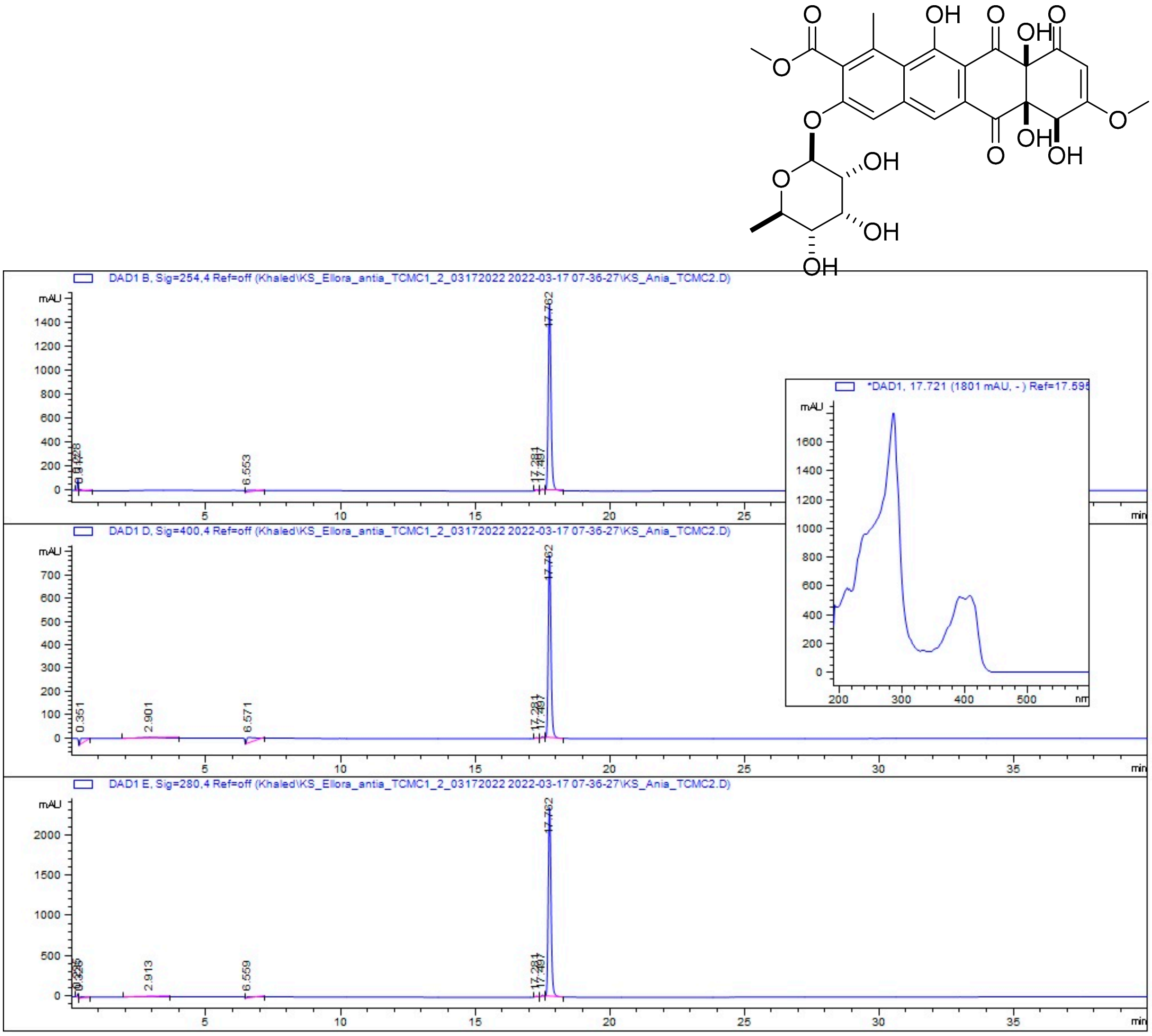

Figure S19: HPLC-UV/vis analysis of 8-demethyl-8-O-β-D-allosyl-TCMC. HPLC-conditions: solvent A: H_2O/0.1% FA; solvent B: CH_3CN; flow rate: 0.5 mL min^{-1}; 0-30 min, 5-100% B; 30-35 min, 100% B; 35-36 min, 100-5% B; 36-40 min, 5% B; Phenomenex NX-C18 column (250 × 4.6 mm, 5 μm); 254 nm, 280 nm, 400 nm. UV-vis inset of full wavelength scan (190-600 nm). Previously reported in Tirkkonen et al., 2023 [7].

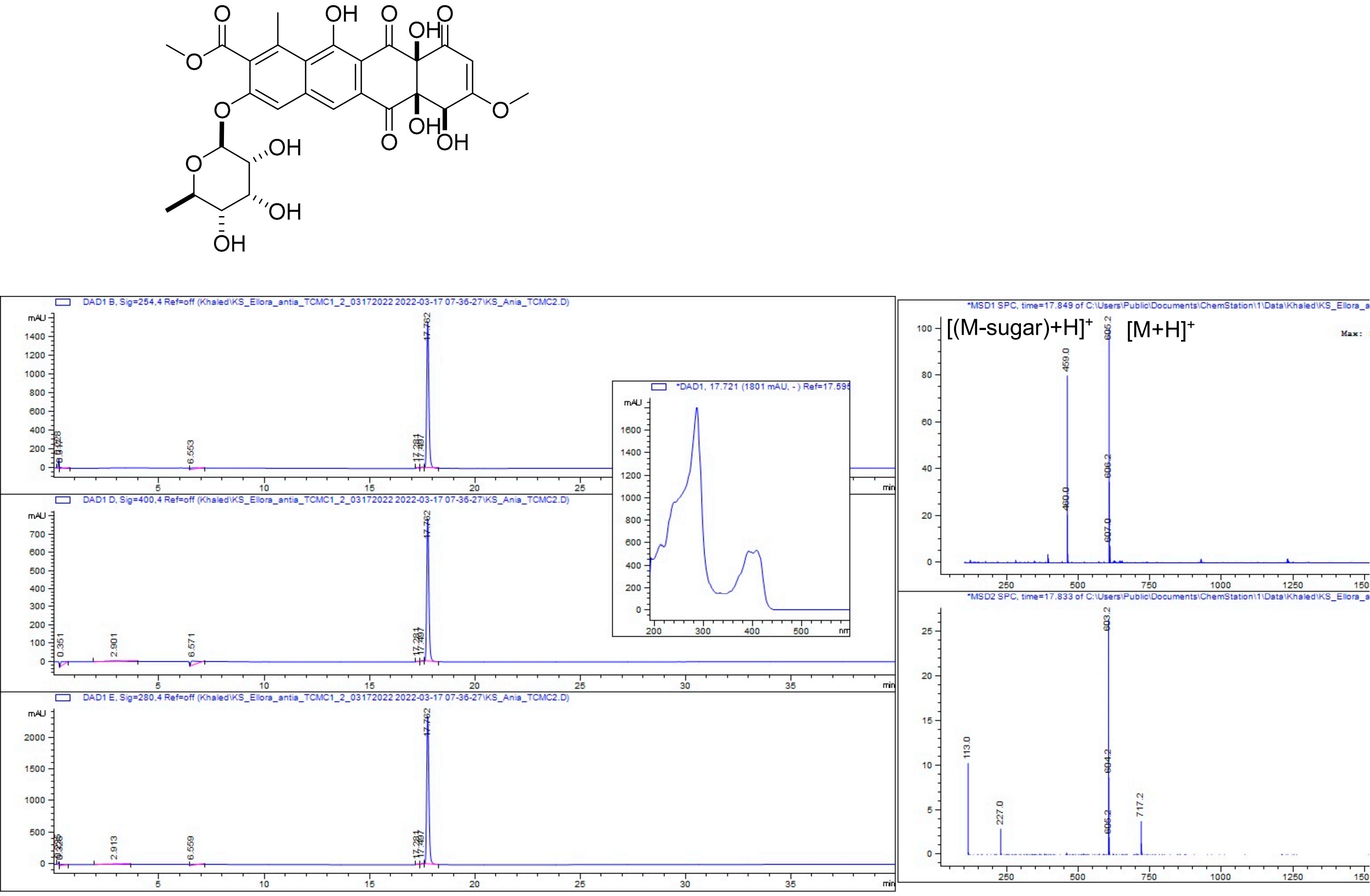

Figure S20. HPLC-MS analysis of 8-demethyl-8-O-β-D-allosyl-TCMC. HPLC-conditions: solvent A: H2O/0.1% FA; solvent B: CH3CN; flow rate: 0.5 mL min-1; 0-30 min, 5-100% B; 30-35 min, 100% B; 35-36 min, 100-5% B; 36-40 min, 5% B; Phenomenex NX-C18 column (250 × 4.6 mm, 5 µm); 254 nm. UV-vis inset of full wavelength scan (190-600 nm). Previously reported in Tirkkonen et al., 2023 [7].

References

1. Sambrook J, W Russell D (2001) Molecular Cloning: A Laboratory Manual. *Cold Spring Harbor Laboratory Press, Cold Spring Harbor, NY*, :999. http://books.google.com/books?id=YTxKwWUiBeUC&printsec=frontcover%5Cnpapers2://publication/uuid/BBBF5563-6091-40C6-8B14-06ACC3392EBB
2. MacNeil DJ, Gewain KM, Ruby CL, Dezeny G, Gibbons PH, MacNeil T (1992) Analysis of Streptomyces avermitilis genes required for avermectin biosynthesis utilizing a novel integration vector. *Gene*, 111(1):61–68. https://doi.org/10.1016/0378-1119(92)90603-m
3. Kieser T, Bibb MJ, Buttner MJ, Chater KF, Hopwood DA (2000) Practical Streptomyces Genetics. *John Innes Centre Ltd.*, :529. https://doi.org/10.4016/28481.01
4. Nybo SE, Shabaan KA, Kharel MK, Sutardjo H, Salas JA, Méndez C, Rohr J (2012) Ketoolivosyl-tetracenomycin C: A new ketosugar bearing tetracenomycin reveals new insight into the substrate flexibility of glycosyltransferase ElmGT. *Bioorganic and Medicinal Chemistry Letters*, 22(6):2247–2250. https://doi.org/10.1016/j.bmcl.2012.01.094
5. Nguyen JT, Riebschleger KK, Brown K V, Gorgijevska NM, Nybo SE (2022) A BioBricks toolbox for metabolic engineering of the tetracenomycin pathway. *Biotechnology Journal*, 17(3):2100371. https://doi.org/10.1002/BIOT.202100371
6. Mazodier P, Petter R, Thompson C (1989) Intergeneric conjugation between Escherichia coli and Streptomyces species. *Journal of Bacteriology*, 171(6):3583–3585. https://doi.org/10.1111/j.1574-6968.1997.tb13882.x
7. Tirkkonen H, Brown K V, Niemczura M, Faudemer Z, Brown C, Ponomareva L V, Helmy YA, Thorson JS, Nybo SE, Metsä-Ketelä M, Shaaban KA Engineering BioBricks for Deoxysugar Biosynthesis and Generation of New Tetracenomycins. https://doi.org/10.1021/acsomega.3c02460
8. Poku RA, Jones KJ, Baren M Van, Alan JK, Amissah F (2020) Diclofenac Enhances Docosahexaenoic Acid-Induced Apoptosis in Vitro in Lung Cancer Cells. *Cancers*, 12(9):1–19. https://doi.org/10.3390/CANCERS12092683
9. Poku R, Amissah F, Alan JK (2023) PI3K Functions Downstream of Cdc42 to Drive Cancer phenotypes in a Melanoma Cell Line. *Small GTPases*, 14(1):1–13. https://doi.org/10.1080/21541248.2023.2202612
10. Friedrich J, Seidel C, Ebner R, Kunz-Schughart LA (2009) Spheroid-based drug screen: considerations and practical approach. *Nature Protocols 2009 4:3*, 4(3):309–324. https://doi.org/10.1038/nprot.2008.226
11. Qiao X, Gan M, Wang C, Liu B, Shang Y, Li Y, Chen S (2019) Tetracenomycin X Exerts Antitumour Activity in Lung Cancer Cells through the Downregulation of Cyclin D1. *Marine Drugs*, 17(1):63. https://doi.org/10.3390/md17010063
12. Asong GM, Amissah F, Voshavar C, Nkembo AT, Ntantie E, Lamango NS, Ablordeppey SY (2020) A Mechanistic Investigation on the Anticancer Properties of SYA013, a Homopiperazine Analogue of Haloperidol with Activity against Triple Negative Breast Cancer Cells. *ACS omega*, 5(51):32907–32918. https://doi.org/10.1021/ACSOMEGA.0C03495
13. Aubry C, Pernodet JL, Lautru S (2019) Modular and integrative vectors for synthetic biology applications in Streptomyces spp. *Applied and Environmental Microbiology*, 85(16)https://doi.org/10.1128/AEM.00485-19

14. Wezel GP Van, Krabben P, Traag BA, Keijser BJF, Kerste R, Vijgenboom E, Heijnen JJ, Kraal B (2006) Unlocking Streptomyces spp. for use as sustainable industrial production platforms by morphological engineering. *Applied and Environmental Microbiology*, 72(8):5283–5288. https://doi.org/10.1128/AEM.00808-06
15. Flett F, Mersinias V, Smith CP, ’ FF, Mersinias V, Smith CP (1997) High efficiency intergeneric conjugal transfer of plasmid DNA from *Escherichia coli* to methyl DNA-restricting Streptomycetes. *FEMS Microbiology Letters*, 155(2):223–229. https://doi.org/10.1016/S0378-1097(97)00392-3
16. Wang R, Nguyen J, Hecht J, Schwartz N, Brown K, Ponomareva L, Niemczura M, Dissel D van, Wezel G van, Thorson J, Metsä-Ketelä M, Shaaban K, Nybo S A BioBricks Metabolic Engineering Platform for the Biosynthesis of Anthracyclinones in <italic toggle="yes">Streptomyces coelicolor</italic>. *ACS Synthetic Biology*, 11(12):4193–4209. https://doi.org/10.1021/acssynbio.2c00498
