# Supplementary material for: Tetracenomycin Aglycones Primarily Inhibit Cell Growth and Proliferation in Mammalian Cancer Cell Lines
Source: Appl Sci (Basel). Author manuscript; Available in PMC 2026 Jun 16. (PMC13267936; doi:10.3390/app152211985)
Supplement: Supplementary Material [file NIHMS2183971-supplement-Supplementary_Material.zip › Supplementary File S2.pdf]

Expanded Materials and Methods:

Bacterial Strains and Growth Conditions

E. coli DH5α and *E. coli* ET12567 were grown at 37°C in L.B. broth or L.B. agar as previously described [1]. *E. coli* DH5α was used for plasmid propagation and subcloning, while *E. coli* ET12567/pUZ8002 was used as the conjugation donor host for mobilizing expression vectors into *Streptomyces coelicolor* M1152Δ*matAB* as previously described [2]. When appropriate, ampicillin (100 μg mL^{-1}), kanamycin (25 μg mL^{-1}), apramycin (25 μg mL^{-1}), viomycin (30 μg mL^{-1}), hygromycin (50 μg mL^{-1}), and nalidixic acid (35 μg mL^{-1}) were supplemented to media to select for recombinant microorganisms.

Streptomyces coelicolor M1152Δ*matAB* and derivative strains were routinely maintained on Soya-Mannitol Flour (S.F.M.) agar supplemented with 10 mM $MgCl_2$ and International Streptomyces Project medium #4 (ISP4) (BD Difco) at 30°C as described previously [3]. For liquid culturing, *Streptomyces coelicolor* M1152Δ*matAB*::cos16F4iE derivative strains were grown in T.S.B. media for the production of seed culture, modified SG-TES liquid medium, and liquid minimal media with supplementation [3, 4]. All media and reagents were purchased from Thermo-Fisher Scientific. Tetracenomycins were isolated and analyzed via HPLC-MS as described previously [5]. Strains are described in Table S2 in Supporting Information.

General genetic manipulations

Routine genetic cloning and plasmid manipulation were carried out in *E. coli* DH5alpha (New England Biolabs). *E. coli* ET12567/pUZ8002 was used as the host for intergeneric conjugation with *Streptomyces coelicolor* as previously described [3]. *E. coli* was transformed with plasmid DNA via chemically competent heat-shock transformation as described previously [1]. Plasmid

DNA was isolated via the Wizard® Plus SV Minipreps DNA Purification System following the manufacturer's protocols (Promega). All molecular biology reagents and enzymes used for plasmid construction were purchased from New England Biolabs. The conjugation donor host *E. coli* ET12567/pUZ8002 was transformed with constructs for mobilization into *Streptomyces coelicolor* M1146::cos16F4iE, as previously described [6]. For each transformation, 9 to 12 independent exconjugants were plated to DNA plates supplemented with antibiotics and grown for 4 to 5 days until the formation of vegetative mycelium. Plasmids are described in Table S1 in Supporting Information.

Small-scale production and Analysis of Tetracenomycins

Tetracenomycin C (CAS 71135-22-3, NSC 309451), tetracenomycin X (NSC 376882), and elloramycinone (NSC 362774) were obtained as pure compounds from the National Cancer Institute Developmental Therapeutics Program (Bethesda, MD). 6-hydroxy-tetracenomycin C was obtained from Dr. Khaled Shaaaban at the University of Kentucky Center for Pharmaceutical Research and Innovation (UK-CPRI, Lexington, KY). Fermentations of *S. olivaceus* Tü 2353*, Streptomyces glaucescens*, and *S. coelicolor* M1146::cos16F4iE and derivative strains were carried out in triplicate 50 mL shake flasks of SG media for 5–7 days as previously described.[12] For analytical scale analyses, 25 mL of culture was extracted with 25 mL of ethyl acetate + 0.1% formic acid in a 50 mL conical tube. The organic phase was collected and concentrated in a rotary evaporator. The red-orange residue was resuspended in 4 mL of methanol and filtered, and a 10 µL was sampled via HPLC-MS analysis. Analyses and quantification of tetracenomycins were carried out on an Agilent 1260 Infinity II LC/MSD iQ single quadrupole instrument. In brief, 10 µL of the sample was injected via an autosampler onto the sample loop and was separated on a Poroshell 120 Phenyl-Hexyl column (ID 2.7 µm, 4.6 mm × 100 mm) and was analyzed in gradients of solvent A (0.1% formic acid in water) and

solvent B (0.1% formic acid in acetonitrile). The HPLC program used a constant flow rate of 0.5 mL/min and the following gradient steps: 0 min, 95% solvent A and 5% solvent B; 0–10 min, 95% solvent A and 5% solvent B to 5% solvent A and 95% solvent B; 10–13 min, held at 5% solvent A and 95% solvent B; 13.1 min, re-equilibrate to 95% solvent A and 5% solvent B; and 13.1–15.1 min, 95% solvent A and 5% solvent B. The diode array detector (DAD) was set to monitor UV–vis absorbance at 290 and 410 nm. The ESI-MS was set to scan from 200 to 1000 *m*/*z* fragments in positive and negative ionization modes.

Scale-up fermentation and isolation of tetracenomycins

For scale-up studies of elloramycin, 8-demethyl-tetracenomycin C, tetracenomycin C, and 8-*O*-D-allosyl-tetracenomycin C, the producing strains were grown on Soya-Mannitol Flour (SFM) plates for 3 to 4 days until well sporulated (with antibiotics for recombinant strains, as appropriate). A 3 cm^2 agar slug with well-sporulated mycelia was inoculated into a baffled 250 mL Erlenmeyer flask with 100 mL of tryptic soy broth medium and was fermented in an orbital shaker at 30 degrees Celsius at 200 RPM for 24 hours to generate a seed culture. 5 L of SG-TES production media was prepared in 100 mL aliquots in 250 mL Erlenmeyer flasks and sterilized. From the seed culture, a 1% v/v inoculum was used to inoculate each of 50 flasks, which were then fermented for 5 days as described above. The resulting production cultures were centrifuged in an Eppendorf 5810R benchtop centrifuge at 3,000 RPM for 10 minutes to separate the mycelia from the fermentation broth. The fermentation broth was mixed with 80 g XAD-7 resin and stirred for 6 hours to adsorb the tetracenomycins. The mycelia was separately extracted with 3 x 1 L ethyl acetate and dried down. The XAD-7 resin was filtered, washed, and the compounds were eluted with 1 L methanol. The organic extracts were combined, concentrated *in vacuo*, and back-extracted with ethyl acetate to obtain the crude tetracenomycins. The resulting extract was dissolved in 9:1 chloroform/methanol, dry-loaded on

a 25 g silica cartridge, and fractionated on a silica cartridge (24 g silica RediSep Rf Gold) on a Teledyne Combiflash 100 instrument using a gradient of chloroform to 9:1 chloroform/methanol at a flow rate of 30 mL/min over 15 min. The resulting fractions were dried down to afford the purified metabolites. The tetracenomycins were evaluated for purity via HPLC-MS (Supporting Information).

Cell culture studies

To determine the potential efficacy of tetracenomycins, a panel of 5 cancer cell lines purchased from American Type Culture Collection (Manassas, VA. U.S.A.) was selected: Human lung adenocarcinoma cells (A549), thyroid cancer cells (MDA-T41), neuroblastoma cells (SK-N-AS), urinary bladder cancer cells (T-24), and acute myeloblastic leukemia cells (Kasumi-1). These cell lines were selected to assess whether the tetracenomycins are potentially effective against a wide range of tumors or not(1). In addition, this panel of cell lines is fundamental to determine whether tetracenomycins will be broadly effective in preventing proliferation across an array of mutations presented by the different cell lines or, more precisely, against specific mutations.

Cell culture media (Hams' F-12K, RPMI 1640, DMEM, McCoy's 5A), and fetal bovine serum (FBS) were also purchased from American Type Culture Collection (Manassas, VA. U.S.A.). Antibiotic mixture (penicillin-streptomycin) was obtained from Life Technologies; Thermo Fisher (Grand Island, NY. U.S.A.). Hank's balanced salt solution, Phosphate buffered saline solution, Resazurin, daunorubicin (DNRB), ApopNexin Annexin V FITC Apoptosis Kit, cell cycle analysis kit, and DMSO were purchased from (Sigma Aldrich, St. Louis, MO. U.S.A.). 8-demethyl-tetracenomycin C (8-DMTC), 6-hydroxy-tetracenomycin C (6-OHT), Elloramycin (ELM), and D-allosyl-tetracenomycin C (DALO-TCMC) were isolated as previously described [7]. Tetracenomycin C (TCMC), Tetracenomycin X (TCMX), and elloramycinone (ELMO) were obtained from the National Cancer Institute Developmental Therapeutics Program (D.T.P.).

Cell culture

A549 cells, MDA-T41 cells, SK-N-AS cells, and T-24 cells were cultured in Ham's F-12K, RPMI 1640, DMEM, and McCoy's 5A media, respectively. All media were supplemented with 10% FBS and 1% penicillin-streptomycin. Kasumi-1 cells cultured in RPMI 1640 media supplemented with 20% FBS and 1% penicillin-streptomycin. The cells were incubated in a humidified incubator with an atmosphere of 95% CO_2 set at 37 °C and sub-cultured at approximately 80-90% confluent. Unless stated otherwise, all assays were performed with experimental media containing 5% FBS.

Cell viability studies

Cultured cells were suspended in 100 μL media were seeded at a density of 1×10^4 cells per well in 96-well plates and allowed to attach over 24 h in media containing 5% FBS. Cell lines were treated with 0 – 100 μM of tetracenomycins (TCMX, TCMC, and 8-DMTC) in methanol. A549, MDA-T41, SK-N-AS, and T-24 cells were treated with ELM, ELMO, and DALO-TCMC (0 – 100 μM). MDA-T41 cells were also treated with 6-OHT (0 – 100 μM). Control cells were treated with equivalent volumes of methanol, and positive control cells were treated with daunorubicin (DNRB; 0 – 50 μM). The treatments were repeated in 24-hour increments for the 48-hour assay. To determine and quantify the effect of the compounds on cell viability, a resazurin reagent metabolized by live cells to the fluorescent resorufin product was used as previously described [8, 9]. Briefly, 20 μL of 0.02% resazurin reagent prepared in PBS was added to each well and incubated for 2-3 h. The fluorescence was measured at 560 nm excitation wavelength and a detection wavelength of 590 nm using the Cytation 3 Multi-Mode Reader from BioTek (Winooski, VT. U.S.A.).

To better observe drug response characteristics in a system that closely simulates the in vivo situation of the tumor, the effect of tetracenomycins was studied using *3D spheroid culture*, as previously described [10, 11]. A549 and MDA-T41 cells were plated 10^4 cells per well in a 96-well ultralow-attachment Lipidure-coat U-shaped clear-bottom plate (Thermo Fisher, Grand Island, NY. U.S.A.). The cells were incubated at 37 °C /5% CO_2, and spheroids were formed after 24 h. The formed spheroids were then exposed to TCMC, TCMX, and ELM (0-100 μM), and the treatment was repeated after 24 h for 48 h. Bright-field images of the effects of the drugs on the spheroids were captured using Cytation 1 Cell Imaging Multi-Mode Reader. Cell viability was determined using CellTiter-Glo® 3D reagent (Promega, WI). Using GraphPad Prism 9 software (San Diego, CA, U.S.A.), cell viability was expressed as the percentage of the fluorescence in the treated cells relative to that of the controls, and the IC_{50} values were determined from the plots of the non-linear regression of the logs of tetracenomycin concentrations.

Colony Forming Assays

Cultured A549, MDA-T41, and T-24 cells were seeded at a density of 5×10^4 cells per well in 12-well plates and incubated overnight at 37 °C in 5% CO_2/95% humidified air. The cells were treated with TCMX, TCMX, or ELM at 0, 5, 10, and 20 μM for 72 h. The cells were washed, trypsinized, counted, replated at 500 or 1000 cells/well, and incubated in fresh medium containing 10% (*v/v*) fetal bovine serum for 10–14 days as previously described [12]. The resulting colonies were fixed with a 7:1 (*v/v*) mixture of methanol and acetic acid, stained with 1% crystal violet, and the number of colonies containing > 50 cells was counted using ImageJ (https://imagej.nih.gov/ij/).

Apoptosis Assay

Flow cytometry analysis was conducted for the apoptosis assay. Cultured A549 or MDA-T41 cells seeded at 10^5 cells per mL in 6-well plates were treated with TCMC or TCMX (0–10 μM) for 48 h.

Apoptosis was determined using the ApopNexin FITC Apoptosis Detection Kit (Millipore Sigma, MO, U.S.A.) according to the manufacturer's protocol. Briefly, treated cells were harvested, washed twice with cold PBS, and suspended in a binding buffer. Annexin-V-FITC and propidium iodide were added to the cell suspensions and incubated for 15 min at room temperature in the dark. The analysis was performed at Van Andel Institute Flow Cytometry Core (Grand Rapids, MI) on a CytoFLEX instrument (Beckman Coulter, CA, U.S.A.).

Data Analysis

All experiments were performed with at least three independent biological replicates of three to four technical replicates each. The exact numbers of biological replicates (n) are noted in the figure legends. Treatment groups were compared with control groups using either an unpaired t-test or one-way analysis of variance (ANOVA) where appropriate, with Dunnet's post hoc test for multiple comparisons using GraphPad Prism 9 software. Differences were considered statistically significant and denoted as (∗) when $p<0.05$.

References

1. Sambrook J, W Russell D (2001) Molecular Cloning: A Laboratory Manual. *Cold Spring Harbor Laboratory Press, Cold Spring Harbor, NY*, :999. http://books.google.com/books?id=YTxKwWUiBeUC&printsec=frontcover%5Cnpapers2://publication/uuid/BBBF5563-6091-40C6-8B14-06ACC3392EBB
2. MacNeil DJ, Gewain KM, Ruby CL, Dezeny G, Gibbons PH, MacNeil T (1992) Analysis of Streptomyces avermitilis genes required for avermectin biosynthesis utilizing a novel integration vector. *Gene*, 111(1):61–68. https://doi.org/10.1016/0378-1119(92)90603-m
3. Kieser T, Bibb MJ, Buttner MJ, Chater KF, Hopwood DA (2000) Practical Streptomyces Genetics. *John Innes Centre Ltd.*, :529. https://doi.org/10.4016/28481.01
4. Nybo SE, Shabaan KA, Kharel MK, Sutardjo H, Salas JA, Méndez C, Rohr J (2012) Ketoolivosyl-tetracenomycin C: A new ketosugar bearing tetracenomycin reveals new insight into the substrate flexibility of glycosyltransferase ElmGT. *Bioorganic and Medicinal Chemistry Letters*, 22(6):2247–2250. https://doi.org/10.1016/j.bmcl.2012.01.094
5. Nguyen JT, Riebschleger KK, Brown K V, Gorgijevska NM, Nybo SE (2022) A BioBricks toolbox for metabolic engineering of the tetracenomycin pathway. *Biotechnology Journal*, 17(3):2100371. https://doi.org/10.1002/BIOT.202100371
6. Mazodier P, Petter R, Thompson C (1989) Intergeneric conjugation between Escherichia coli and Streptomyces species. *Journal of Bacteriology*, 171(6):3583–3585. https://doi.org/10.1111/j.1574-6968.1997.tb13882.x
7. Tirkkonen H, Brown K V, Niemczura M, Faudemer Z, Brown C, Ponomareva L V, Helmy YA, Thorson JS, Nybo SE, Metsä-Ketelä M, Shaaban KA Engineering BioBricks for Deoxysugar Biosynthesis and Generation of New Tetracenomycins. https://doi.org/10.1021/acsomega.3c02460
8. Poku RA, Jones KJ, Baren M Van, Alan JK, Amissah F (2020) Diclofenac Enhances Docosahexaenoic Acid-Induced Apoptosis in Vitro in Lung Cancer Cells. *Cancers*, 12(9):1–19. https://doi.org/10.3390/CANCERS12092683
9. Poku R, Amissah F, Alan JK (2023) PI3K Functions Downstream of Cdc42 to Drive Cancer phenotypes in a Melanoma Cell Line. *Small GTPases*, 14(1):1–13. https://doi.org/10.1080/21541248.2023.2202612
10. Friedrich J, Seidel C, Ebner R, Kunz-Schughart LA (2009) Spheroid-based drug screen: considerations and practical approach. *Nature Protocols 2009 4:3*, 4(3):309–324. https://doi.org/10.1038/nprot.2008.226
11. Qiao X, Gan M, Wang C, Liu B, Shang Y, Li Y, Chen S (2019) Tetracenomycin X Exerts Antitumour Activity in Lung Cancer Cells through the Downregulation of Cyclin D1. *Marine Drugs*, 17(1):63. https://doi.org/10.3390/md17010063
12. Asong GM, Amissah F, Voshavar C, Nkembo AT, Ntantie E, Lamango NS, Ablordeppey SY (2020) A Mechanistic Investigation on the Anticancer Properties of SYA013, a Homopiperazine Analogue of Haloperidol with Activity against Triple Negative Breast Cancer Cells. *ACS omega*, 5(51):32907–32918. https://doi.org/10.1021/ACSOMEGA.0C03495
13. Aubry C, Pernodet JL, Lautru S (2019) Modular and integrative vectors for synthetic biology applications in Streptomyces spp. *Applied and Environmental Microbiology*, 85(16)https://doi.org/10.1128/AEM.00485-19

14. Wezel GP Van, Krabben P, Traag BA, Keijser BJF, Kerste R, Vijgenboom E, Heijnen JJ, Kraal B (2006) Unlocking Streptomyces spp. for use as sustainable industrial production platforms by morphological engineering. *Applied and Environmental Microbiology*, 72(8):5283–5288. https://doi.org/10.1128/AEM.00808-06
15. Flett F, Mersinias V, Smith CP, ’ FF, Mersinias V, Smith CP (1997) High efficiency intergeneric conjugal transfer of plasmid DNA from *Escherichia coli* to methyl DNA-restricting Streptomycetes. *FEMS Microbiology Letters*, 155(2):223–229. https://doi.org/10.1016/S0378-1097(97)00392-3
16. Wang R, Nguyen J, Hecht J, Schwartz N, Brown K, Ponomareva L, Niemczura M, Dissel D van, Wezel G van, Thorson J, Metsä-Ketelä M, Shaaban K, Nybo S A BioBricks Metabolic Engineering Platform for the Biosynthesis of Anthracyclinones in <italic toggle="yes">Streptomyces coelicolor</italic>. *ACS Synthetic Biology*, 11(12):4193–4209. https://doi.org/10.1021/acssynbio.2c00498
